# Machine Learning-Based Prediction of Transthyretin-Binding Activity and Its Application to Screening Food-Derived Compounds for ATTR Amyloidosis

**DOI:** 10.3390/ph19081295

**Published:** 2026-08-16

**Authors:** Yuma Iwashita, Yoshihiro Uesawa

**Affiliations:** Department of Medical Molecular Informatics, Meiji Pharmaceutical University, Tokyo 204-8588, Japan

**Keywords:** transthyretin amyloidosis, wild-type ATTR, transthyretin stabilizer, machine learning, in silico screening, food-derived compounds, polyphenols

## Abstract

**Background/Objectives**: Transthyretin amyloidosis (ATTR) is a progressive disease caused by the dissociation of the transthyretin (TTR) tetramer, leading to amyloid fibril formation. Although pharmacological stabilizers have been developed, preventive strategies for wild-type ATTR (ATTRwt) have not been established. This study developed a computational model to predict TTR-binding activity from chemical structures and applied it to the exploratory prioritization of food-derived compounds. **Methods**: A machine learning model was constructed using TTR–8-anilino-1-naphthalenesulfonic acid displacement assay data from the Tox24 Challenge. An integrated dataset compiled from multiple literature sources was used to compare predicted TTR-binding activity with amyloid fibril formation inhibition, and the trained model was applied to the PhytoHub database. **Results**: The model achieved a root mean square error of 21.34 and a mean absolute error of 15.9 percentage points on an external test dataset, a performance comparable to that of the top-scoring models in the Tox24 Challenge. The predicted TTR-binding activity showed positive correlations with inhibition of amyloid fibril formation, Pearson’s r = 0.673 and Spearman’s ρ = 0.602. The model prioritized 39 compounds with high predicted binding activity, predominantly polyphenols. **Conclusions**: These results suggest that the proposed in silico method may be useful for the exploratory prioritization of food-derived compounds with potential relevance to TTR stabilization.

## 1. Introduction

Transthyretin amyloidosis (ATTR) is a form of amyloidosis caused by transthyretin (TTR), broadly classified into the hereditary (ATTRv) and wild-type (ATTRwt) forms [1]. In ATTRv, clinical manifestations, e.g., familial amyloidotic polyneuropathy and familial amyloid cardiomyopathy, are well recognized [2,3]. Conversely, ATTRwt, formerly known as senile systemic amyloidosis, has also been clinically characterized [4].

TTR is a transport protein in plasma and cerebrospinal fluid involved in the distribution of thyroxine and retinol, the latter occurring via its association with retinol-binding protein [5].

Amyloid formation from TTR proceeds via an initial dissociation of the tetrameric structure [6], and this tetramer dissociation represents the rate-limiting step in pathogenesis, after which the released monomers undergo partial structural denaturation, becoming amyloidogenic [7]. Ultimately, these monomers aggregate to form amyloid fibrils and amorphous deposits [8].

In ATTRwt, amyloid deposition in the myocardium leads to impaired diastolic function and progressive congestive heart failure. Clinically, this disease predominantly manifests as diastolic dysfunction, frequently presenting as heart failure with preserved ejection fraction. In addition, carpal tunnel syndrome and spinal stenosis may precede cardiac symptoms [9].

Based on this pathogenic mechanism, pharmacological agents that stabilize TTR and slow the progression of amyloid formation have been approved. A representative drug is tafamidis, indicated for ATTRwt cardiomyopathy, ATTRv cardiomyopathy, and early-stage ATTRv polyneuropathy [1]. However, the treatment cost is substantial. For example, the annual list price of tafamidis in the United States has been reported to reach approximately $225,000 [10]. Thus, long-term continuous therapy can impose significant economic burdens on patients and healthcare insurance systems. Furthermore, no established preventive strategies currently exist for ATTRwt, and knowledge about effective interventions at the presymptomatic stage remains limited. Thus, investigating potential preventive interventions that may reduce the risk of onset or delay the progression of ATTRwt is important.

Research on dietary interventions designed to prevent ATTRwt is limited. However, a previous study demonstrated that arginine stabilizes the TTR tetramer and oral supplementation of 5000 mg per day for 5 days increased the TTR tetramer/monomer ratio, suggesting the potential of supplementation as a preventive intervention [11]. The study suggested that the arginine-mediated TTR stabilization mechanism may involve interactions with aromatic amino acid residues that are abundant in TTR, particularly tryptophan residues.

Furthermore, although specifically targeted at inhibiting disease progression (rather than ATTRwt prevention), epigallocatechin-3-gallate (EGCG), a component of green tea, has been reported to stabilize TTR [12]. An observational clinical study reported that daily consumption of 1.5–2 L of green tea over 12 months resulted in an approximately 13% reduction in left ventricular myocardial mass [13]. TTR stabilization by EGCG is attributed to tetramer stabilization via binding at the TTR dimer–dimer interface, suggesting that food-derived compounds can stabilize TTR [12].

The investigation of natural compounds with TTR-binding properties has been previously reported in the literature [14]. However, these compounds have mainly been examined for their inhibitory effects on TTR amyloid formation. Many food-derived compounds have a history of dietary exposure and may be suitable for investigation in the context of long-term intake. ATTRwt is an age-related disease [15]. Therefore, food-derived compounds may be of interest as a class of compounds for exploratory screening, although their actual preventive relevance requires further experimental and clinical validation. From this perspective, developing a computational screening method based on chemical structural information to prioritize food-derived compounds with potential relevance to TTR-binding and stabilization may be useful.

Ideally, a prediction model based directly on TTR stabilization assays would be the most suitable approach for prioritizing potential TTR stabilizers. However, publicly available TTR stabilization assay datasets are limited in size and consistency, and large-scale datasets containing more than 1000 compounds have not been identified. Therefore, as an indirect approach, this study focused on TTR-binding affinity, which is one of the factors associated with TTR stabilization. Generally, the degree of pharmacological kinetic stabilization of TTR depends on the fraction of TTR tetramers in which a stabilizer occupies at least one binding site [16]. This fraction is determined by the binding affinity of the stabilizer for TTR, its binding affinity for albumin, which is the major plasma protein competing with TTR for stabilizer binding, and the plasma concentrations of the stabilizer, TTR, and albumin. TTR–8-anilino-1-naphthalenesulfonic acid (ANSA) displacement activity, based on the displacement of the fluorescent probe ANSA, has been reported as an indirect indicator of TTR-binding affinity [17]. Accordingly, we developed a model to predict TTR–ANSA displacement activity from chemical structural information using assay data obtained from a structurally diverse set of compounds.

**Figure 1:**
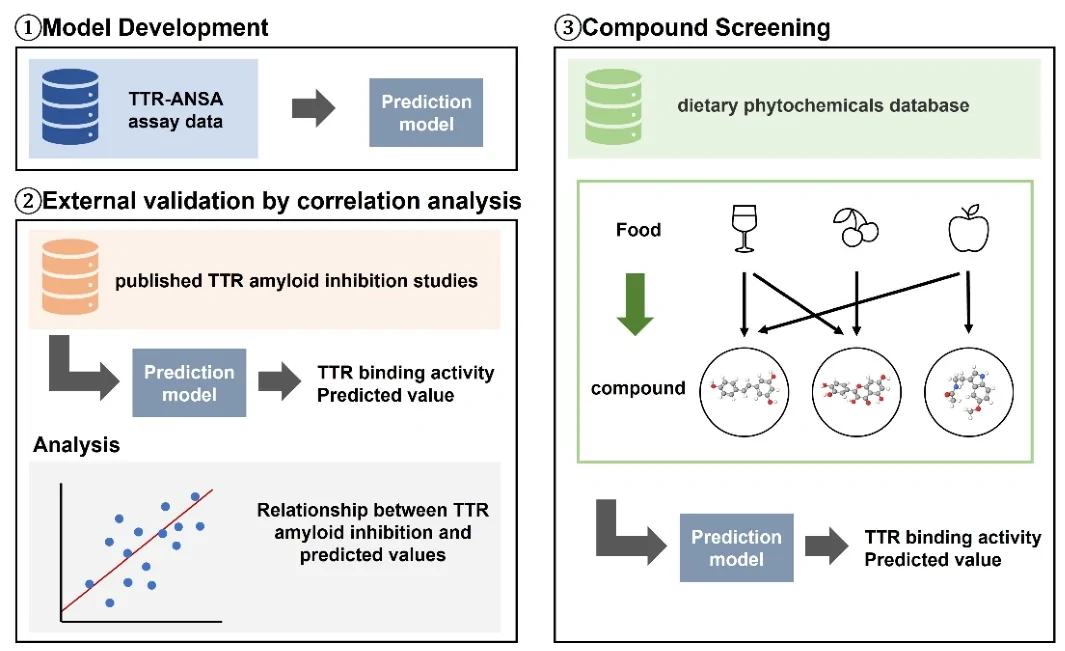
Overview of the analytical workflow. A TTR-binding activity prediction model was constructed using TTR–ANSA assay data. The predicted TTR-binding activity was compared with reported TTR amyloid formation inhibition data collected from the literature to assess their relationship. The constructed model was then applied to a dietary phytochemical database to prioritize compounds with high predicted activity for exploratory screening.

This study aimed to develop an exploratory in silico screening method for prioritizing food-derived compounds with potential relevance to TTR stabilization. First, a displacement assay-based model was constructed to predict TTR-binding activity from chemical structures. Next, to assess whether the predicted binding activity could serve as a screening index for TTR stabilization, we examined its monotonic association with amyloid fibril formation inhibition reported in previous studies under acidic conditions. Finally, the validated model was applied to a food compound database to rank food-derived compounds based on their predicted TTR-binding activity. Overall, this workflow made it possible to identify food-derived compounds with a high predicted TTR-binding activity, providing a basis for exploratory prioritization. Figure 1 shows an overview of the analysis.

## 2. Results

### 2.1. Construction of a TTR-Binding Activity Prediction Model

To construct the TTR-binding activity prediction model, TTR–ANSA displacement activity data published in the 2024 Tox24 Challenge were obtained from the Online Chemical Modeling Environment (OCHEM; https://ochem.eu/static/challenge-data.do on 29 May 2025) [18]. In this study, the train (N = 1012) and leaderboard (N = 200) datasets were combined, and two entries with the same SMILES were merged into a single record, resulting in a training dataset of 1211 unique compounds. The test (N = 300) dataset was used for external validation.

The TTR-binding activity was measured by the ANSA displacement assay as relative activity (%) with respect to thyroxine (T4) [17]. In addition, the range of the TTR-binding activity in the training dataset was −45.0 to 111.1% with a mean of 39.6% and a standard deviation (SD) of 36.1% (Table S1). The TTR-binding activity values did not fall entirely within the 0–100% range, possibly because this assay is based on fluorescence measurements and may be influenced by compound autofluorescence and inner-filter effects. For compounds with autofluorescence, the measured fluorescence intensity may increase independently of ANSA displacement, resulting in apparent activity values below 0%. Conversely, when the measured fluorescence intensity is reduced owing to inner-filter effects, apparent activity values greater than 100% may be obtained.

In addition, the TTR-binding activity values indicate relative ANSA displacement activities, calculated by converting the fluorescence values of each compound tested at 100 µM using the 0% and 100% activity reference conditions established with T4; they do not constitute direct comparisons with T4 under the same concentration conditions [17]. Therefore, the activity values in this study should be interpreted as relative displacement activities defined within the assay system.

#### 2.1.1. Model Selection

To evaluate the effect of molecular descriptor input formats on model performance, the following three formats were compared:

- (a) Without salt/complex processing: molecular descriptors calculated from the original structures;
- (b) After salt/complex processing: molecular descriptors calculated from the processed structures; and
- (c) Integrated format: a combined set of molecular descriptors calculated from structures before and after salt/complex processing.

All molecular descriptors were calculated using the Mordred descriptor package [19]. The predictive performance of these input formats was then evaluated using LightGBM, a gradient-boosting implementation [20].

Model construction was performed in two stages, i.e., feature selection and hyperparameter optimization. Following feature preprocessing and selection, the final numbers of molecular descriptors adopted for formats (a), (b), and (c) were 443, 522, and 820, respectively (Table 1).

Hyperparameter optimization was then performed using these descriptors to determine the optimal set of hyperparameters. Here, the mean root mean square error (RMSE) in fivefold cross-validation (CV) for the training dataset was 23.42, 23.25, and 22.82 for formats (a), (b), and (c), respectively, with format (c) obtaining the highest predictive accuracy (Table 2).

**Figure 2:**
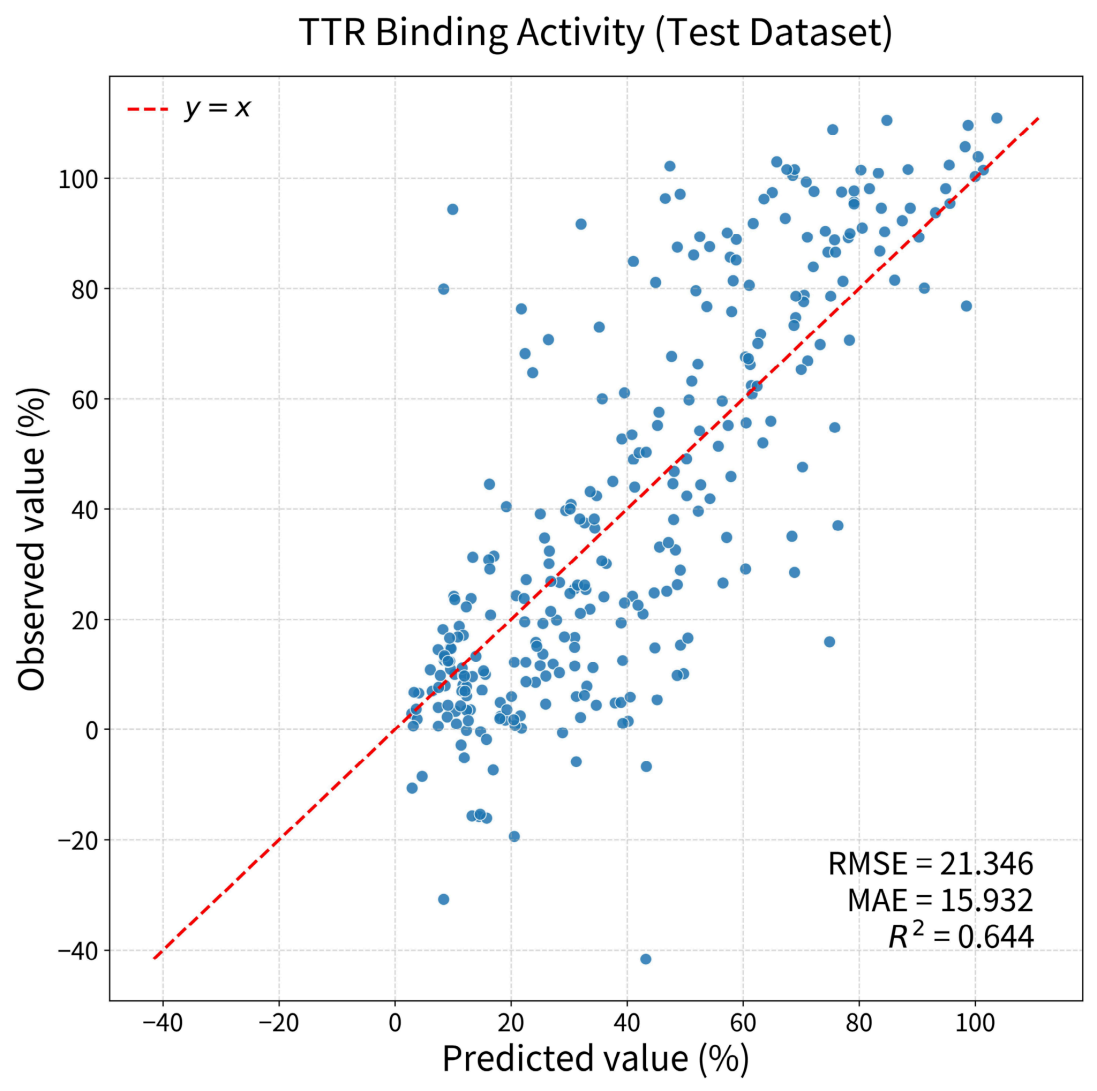
Scatter plot showing the relationship between predicted TTR-binding activity and measured values in the test dataset using the model constructed based on input format (c). Each point represents a compound in the test dataset, and the red dashed line indicates agreement between the predicted and measured values (y=x).

Thus, format (c) was adopted as the final model, and predictions were performed on the test dataset, which was not used during training, yielding an RMSE of 21.34. In addition, the mean absolute error (MAE) and *R^2^* values were 15.9 and 0.64, respectively (Figure 2). In the Tox24 Challenge, the model achieved an RMSE comparable to those of the top-ranking models [21].

#### 2.1.2. Definition of the Applicability Score and Applicability Domain

In this study, Applicability was calculated to evaluate the reliability of predictions, and the Applicability Domain (AD) was defined based on this value [22]. For each compound in the training dataset, the Euclidean distances to the five nearest neighbors $\left(d_{i1},\ldots ,d_{i5}\right)$ in the standardized molecular descriptor space (mean = 0, SD = 1) were calculated. Then, the log-transformed geometric mean of these distances was used as the distance metric $s_{i}$, calculated as follows:

(1) $$s_{i}=\log\left({\left(\prod_{j=1}^{5}d_{ij}\right)}^{1/5}\right)$$

The 95% interval of the $s_{i}$ distribution in the training dataset was used to establish the lower and upper thresholds. For each compound in the test dataset, $s_{i}$ was calculated based on the distances to the five nearest neighbors in the training dataset. Compounds with $s_{i}$ values below the lower threshold were assigned an Applicability value of 1, whereas compounds with $s_{i}$ values above the upper threshold were assigned an Applicability value of 0. Compounds with $s_{i}$ values between the lower and upper thresholds were assigned Applicability values normalized from 1 to 0.

**Figure 3:**
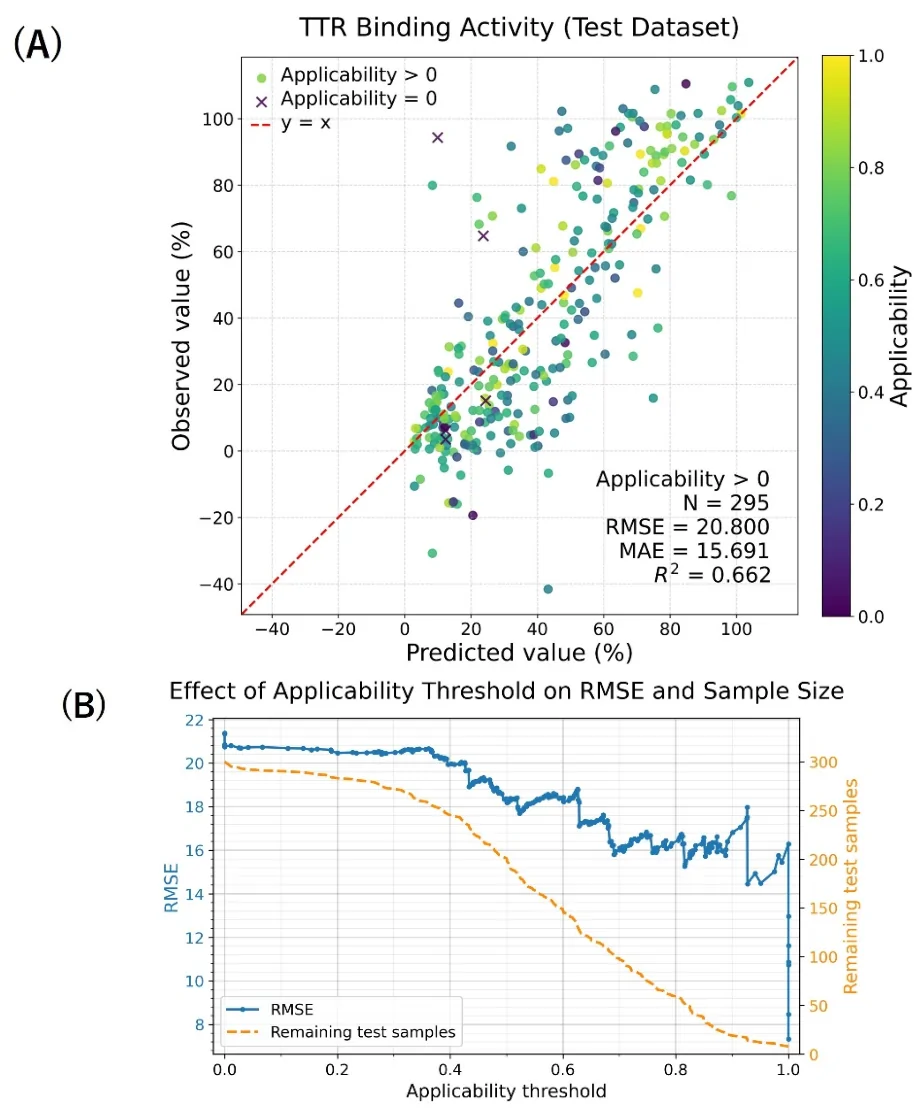
(**A**) Scatter plot of predicted versus observed TTR-binding activity in the test dataset. Circles denote compounds with Applicability > 0, whereas crosses denote compounds with Applicability = 0. Point color represents the Applicability value, and the red dashed line indicates y=x. RMSE, MAE, and $R^{2}$ were calculated after excluding compounds with Applicability =0. (**B**) Changes in RMSE in the test dataset according to the Applicability threshold (blue line) and changes in the number of remaining compounds according to the threshold (orange line).

When Applicability values were calculated for the test compounds, 8 of the 300 compounds received an Applicability value of 1, whereas 5 compounds received an Applicability value of 0 (Figure 3A and Table S2).

When the relationship between the Applicability threshold and RMSE was examined, RMSE generally decreased as the Applicability threshold increased. This trend suggests that samples with higher Applicability values were predicted more accurately. However, at high Applicability thresholds, particularly near 1.0, the number of remaining test samples became markedly small, causing the RMSE values to become unstable. Therefore, although increasing the Applicability threshold improved prediction accuracy to some extent, overly strict thresholds may reduce the reliability of the RMSE estimate owing to the limited number of remaining samples.

Furthermore, excluding compounds with an Applicability value of 0 lowered the RMSE from 21.3 to 20.8. This suggests that Applicability = 0 can serve as a practical criterion for excluding samples that are substantially distant from the descriptor space of the training dataset (Figure 3B). Therefore, compounds with Applicability > 0 were considered to be inside the AD, whereas those with Applicability = 0 were considered to be outside the AD.

#### 2.1.3. SHAP Analysis

**Figure 4:**
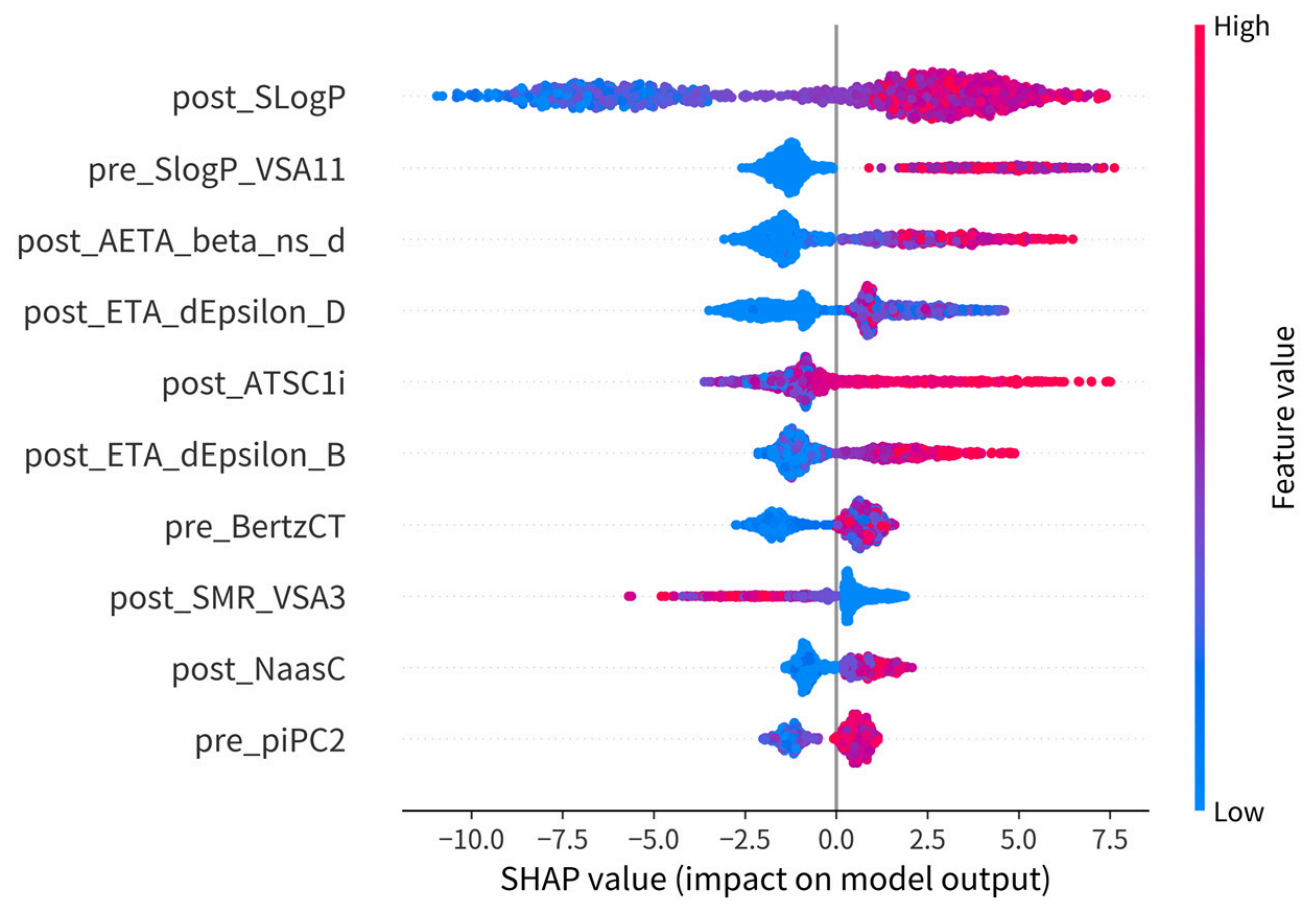
SHAP summary plot showing the contribution of molecular descriptors to predicted values. The horizontal axis represents SHAP values, with positive and negative values indicating contributions to increasing and decreasing the predicted value, respectively. Each point represents an individual compound, and its color indicates the value of the corresponding molecular descriptor, with red and blue indicating high and low values, respectively.

Feature importance was calculated using SHapley Additive exPlanations (SHAP) [23] to interpret the constructed model. Here, the SHAP values were computed using the TreeExplainer implemented in the Python SHAP library, and the entire training dataset was used for evaluation. In this study, feature importance was calculated as the mean absolute SHAP value for each feature and visualized in a summary plot (Figure 4). The prefixes “pre” and “post” denote the Mordred descriptors calculated from the structures before and after salt/complex processing, respectively. The top 10 molecular descriptors in order of importance were post_SLogP, pre_SlogP_VSA11, post_AETA_beta_ns_d, post_ETA_dEpsilon_D, post_ATSC1i, post_ETA_dEpsilon_B, pre_BertzCT, post_SMR_VSA3, post_NaasC, and post_piPC2. The official Mordred description for each descriptor is shown in Table 3.

### 2.2. Correlation Analysis with Amyloid Formation Inhibition Assays

To evaluate whether the predicted TTR-binding activity could serve as an exploratory indicator of TTR stabilization, its association with experimentally reported inhibition of TTR amyloid formation was examined.

Previously reported TTR amyloid formation inhibition assay data were collected from four publications, i.e., Klabunde et al. (2000), Oza et al. (2002), Adamski-Werner et al. (2004), and Palaninathan et al. (2009) [24,25,26,27]. Generally, the assay conditions were consistent across the publications (Table S3). Regarding the presentation of results, Klabunde et al., Oza et al., and Adamski-Werner et al. reported the percentage of fibril formation (% fibril formation) calculated relative to the turbidity observed in the absence of the inhibitor (set at 100%). In contrast, Palaninathan et al. reported the percentage of inhibition (% inhibition) derived from the same turbidity ratio. In the current study, to align the metrics with expectations of a positive correlation with the model’s predicted values, values reported as % fibril formation were converted to inhibition rates by subtracting them from 100.

A total of 12, 13, 27, and 29 data points were obtained from the four publications, respectively. Two compounds overlapped across the publications; thus, the experimental values from the most recently published source were retained. This deduplication process excluded three data points, leaving 78 compounds (Table S4) for analysis.

For these 78 compounds, TTR-binding activity predictions and Applicability assessments were performed using the constructed model. All compounds were classified as within the AD (Applicability > 0), and predicted values were calculated.

**Figure 5:**
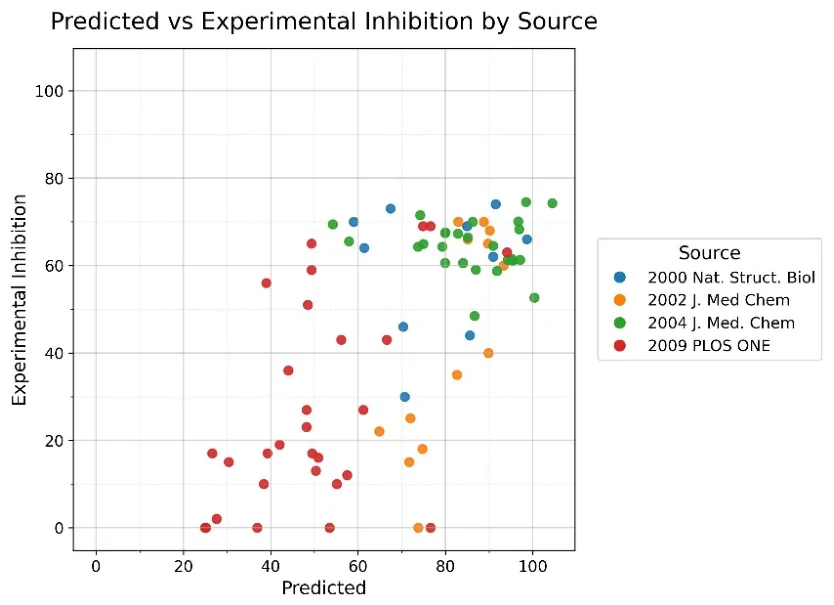
Scatter plot showing the relationship between predicted TTR-binding activity and amyloid formation inhibition rate. Each point represents a compound collected from the literature, and the colors indicate the literature source: blue [24], orange [25], green [26], and red [27].

Correlation coefficients were calculated for each publication and for the integrated dataset consisting of the 78 compounds retained after deduplication. Both Pearson’s correlation coefficient and Spearman’s rank correlation coefficient were determined. The dataset from Oza et al. (2002) [25] showed a strong Pearson correlation and a moderate to strong Spearman correlation. In contrast, the dataset from Palaninathan et al. (2009) [27] showed moderate correlations, whereas the datasets from Klabunde et al. (2000) [24] and Adamski-Werner et al. (2004) [26] showed little to no correlation. When all compounds from the four publications were analyzed together, a moderate positive correlation was observed (Figure 5 and Table 4). To determine whether this pooled association was attributable only to differences across source publications, an additional source-adjusted regression analysis was performed. In the simple regression model, the predicted value was positively associated with observed amyloid inhibition (β = 0.780, *p* = 1.55 × 10^−11^, *R^2^* = 0.452). After source publication was included as a categorical covariate, the coefficient for the predicted value decreased but remained positive and significant (β = 0.553, *p* = 2.25 × 10^−4^, *R^2^* = 0.550), as shown in Table 5. These results suggest that source-dependent heterogeneity contributed to the pooled association, but that the association was not explained only by differences across source publications.

Correlation coefficients between the predicted TTR-binding activity and the amyloid formation inhibition rate were calculated at different Applicability thresholds. Because this screening analysis focused on the consistency of compound ranking rather than on a strictly linear relationship, Spearman’s rank correlation coefficient was used as the primary criterion for threshold selection. Spearman’s correlation remained relatively stable and showed a favorable value at an Applicability threshold of 0.4 (ρ = 0.621). Although Pearson’s correlation coefficient reached a higher value at a threshold of 0.55 (r = 0.733), Applicability ≥ 0.4 was selected as an empirical threshold for exploratory candidate prioritization (Figures S1 and S2).

### 2.3. Screening of Food-Derived Compounds

In this study, the PhytoHub database (version 1.4; https://phytohub.eu/, accessed on 22 November 2025), which provides Simplified Molecular Input Line Entry System (SMILES) notation for its registered compounds, was employed to screen food-derived compounds. The PhytoHub database contains structural information in SMILES notation, structural classification, information on foods from which compounds are derived, and information on compounds that are precursors or metabolites of the corresponding constituents [28].

Note that SMILES information was not assigned to all entries, and it could not be obtained for some compounds. Among the entries lacking SMILES annotations, many were conjugated compounds or compounds described by abstract names, e.g., “Blackberry flavonols,” “Onion flavonols,” and “Blueberry flavonols,” for which the chemical structure could not be uniquely specified. Thus, to investigate the relationship between the missing SMILES information and other annotations, the status of the “Metabolites/Precursor” column was investigated across all PhytoHub entries (Table S5).

As a result, among the entries with missing “Food sources” information (N = 1750), 55.8% (977/1750) included annotations in the “Metabolites/Precursor” column. In contrast, among the entries in which both “Smiles” and “Food sources” were recorded (N = 934), the proportion was only 26.6% (248/934).

Based on these results, 383 entries with missing “Smiles” information and 1750 entries with missing “Food sources” information were excluded, and a final analytical dataset comprising 934 entries was constructed (Table S6).

The compounds in the analytical dataset were assigned to five categories, i.e., “Polyphenols,” “Terpenoids,” “N-containing compounds,” “Miscellaneous phytochemicals,” and “Not Available.” Here, the “Miscellaneous phytochemicals” and “Not Available” categories, which could not be classified unambiguously, were combined as “Others.”

#### Predictions for Food-Derived Compounds

**Figure 6:**
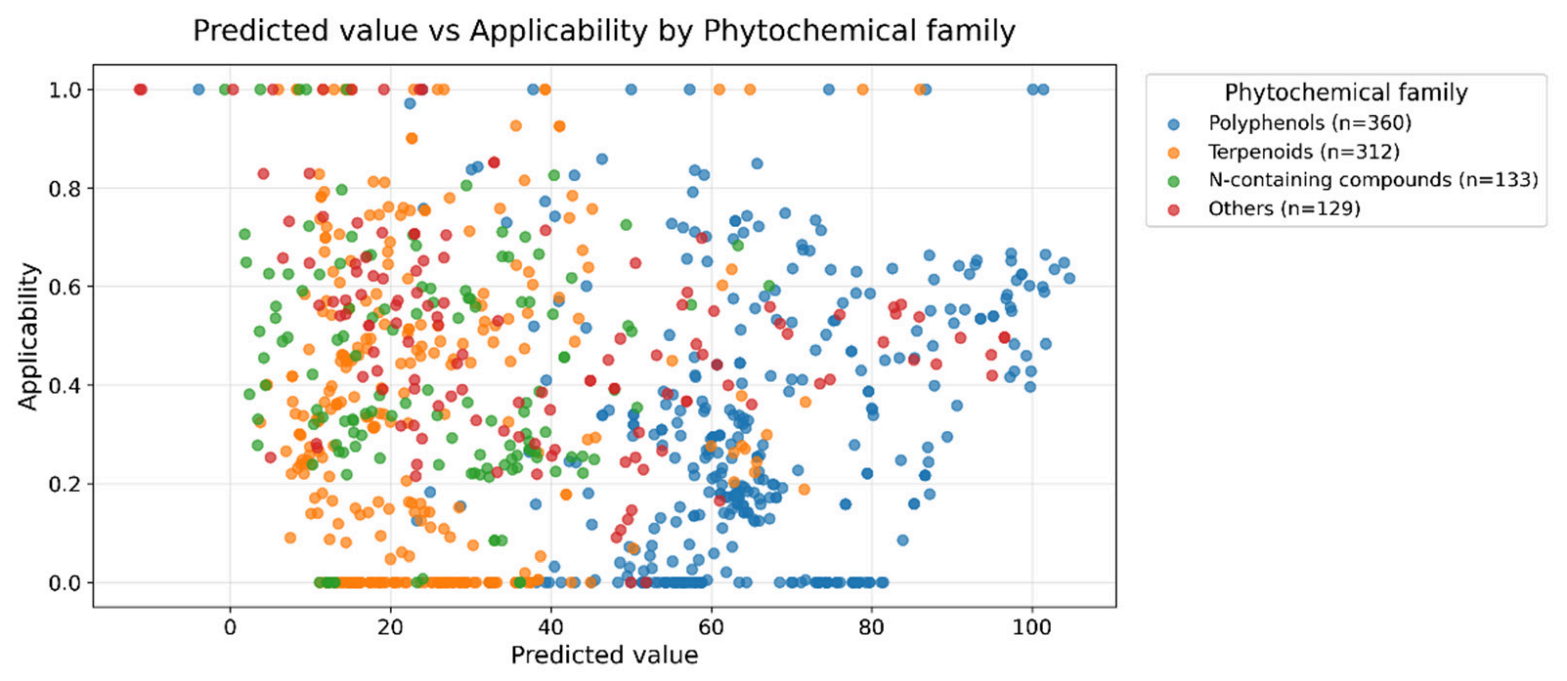
Scatter plot showing predicted TTR-binding activity and Applicability values for food-derived compounds. Each point represents an individual compound in the PhytoHub analytical dataset, and the colors indicate the phytochemical family. The horizontal axis shows predicted TTR-binding activity, and the vertical axis shows the Applicability value. Compounds with Applicability = 0 were considered to be outside the applicability domain.

Predicted values and Applicability values were calculated for the 934 compounds in the analytical dataset. Among all samples, 171 compounds were classified as outside the AD (Applicability = 0), including 76 polyphenols, 84 terpenoids, 9 N-containing compounds, and 2 compounds classified as Others (Table S7). Polyphenols tended to be distributed toward higher predicted values, whereas terpenoids and N-containing compounds tended to be distributed toward lower predicted values (Figure 6). Compounds classified as Others were broadly distributed across the predicted value range.

Among compounds with Applicability ≥ 0.4, which was set as the empirical threshold based on correlation analysis with TTR amyloid formation inhibition data, the compounds were ranked according to their predicted values. To present the upper range of predicted activity, compounds with predicted values of 90% or higher were listed in Table 6. A total of 39 compounds met these criteria, 34 of which were classified as polyphenols. The compound with the highest predicted value was glycitein, which was reported to be present in soybeans. Daidzein, which showed the second-highest predicted value, was reported in PhytoHub to occur in soybeans, soy milk, and red clover (Table S6). Information on compounds with predicted values below 90% and their documented food sources is provided in Tables S6 and S7. Note that the PhytoHub database is partially constructed based on manual registration by domain experts; thus, some compounds may not be clearly assignable to standard scientific classifications [28].

## 3. Discussion

### 3.1. Prediction Model Construction and Accuracy

As described in Section 2.1, format (c), which integrated molecular descriptors calculated from both the original and salt/complex-processed structures, exhibited the highest predictive accuracy among the three feature input formats examined during model construction (Table 2). This suggests that integrating information from different structural states before and after processing may be useful for the target prediction task. However, it could not be clearly determined whether the improvement in performance was attributable to information derived from the salt/complex-processed structures or to the overall effect of expanding the feature representation through integration. Thus, the underlying mechanism remains uncertain. In addition, the RMSE for format (c) was comparable to that of the top-ranking models, corresponding to an estimated seventh-place ranking among the 78 teams participating in the Tox24 Challenge (Figure 2) [21,29]. Furthermore, teams whose prediction scores were not significantly different from those of the winning model, as determined by a paired t-test, were designated as “group winners.” Because the top 11 teams fell into this category, the model constructed in the current study is considered to have similar predictive performance [21].

Despite this comparable performance, the model’s predictions involve some error when used for screening. The MAE, an intuitively interpretable metric, was 15.9, indicating that the predicted values differed from the measured values by an average of approximately 15.9 percentage points. Therefore, when this model is used for screening, the predicted activity values for individual compounds should be interpreted with some uncertainty. Although the model does not predict the activity values of individual compounds with high confidence, it may still be useful as a reference index for the exploratory prioritization of candidate compounds with potentially high activity.

### 3.2. Interpretation by SHAP

Among the top 10 descriptors identified by the SHAP analysis, both pre- and post-salt/complex processing descriptors were included (Figure 4). The top two descriptors were lipophilicity-related, and higher values were associated with higher predicted binding activity (Table 3). This trend is consistent with the hydrophobic environment of the TTR-binding site [30].

In addition to these lipophilicity-related descriptors, several descriptors associated with aromaticity and unsaturation were also identified as important features (Table 3). These descriptors may indirectly reflect structural features of hydrophobic scaffolds and molecular shape. Therefore, the contribution of descriptors related to aromaticity and unsaturation may suggest that the model captured structural tendencies potentially associated with hydrophobic interactions and fit within the TTR-binding channel.

Furthermore, the ETA_dEpsilon_D descriptor reflects hydrogen atoms bonded to heteroatoms (Table 3). This suggests that the model may also account for the contribution of hydrogen-bond-donating functional groups in its predictions. These may include hydroxyl groups, as well as other functional groups such as amino, amide, or carboxyl groups. Among them, hydroxyl groups are particularly plausible in the context of TTR-binding. The TTR thyroxine-binding site contains halogen-binding pockets, and crystallographic studies have indicated that flavonoid hydroxyl groups may form direct or water-mediated hydrogen bonds with residues located within or around these pockets, including Ser117 and Thr119, depending on the ligand orientation [31].

However, because the current model is a statistical model based on molecular descriptors, SHAP analysis does not directly reveal the true binding mechanism or specific ligand–residue interactions. Therefore, the abovementioned interpretations should be considered hypothesis-generating observations about structural factors that may contribute to TTR-binding activity, rather than evidence of specific binding mechanisms. Taken together, these results suggest that the model may have learned structural features associated with TTR-binding activity, including lipophilicity, properties of hydrophobic scaffolds, and hydrogen-bond-donating capacity. These findings further imply that structural modifications affecting lipophilicity, aromatic or unsaturated hydrophobic scaffolds, and hydrogen-bond-donating groups may influence TTR-binding, depending on their position and orientation within the binding site.

### 3.3. Correlation Between Amyloid Formation Inhibition and Predicted TTR-Binding Activity

The TTR–ANSA displacement assay data provided in the Tox24 Challenge were originally intended to evaluate TTR-binding activity, not TTR stabilization activity [18]. Therefore, to determine whether the displacement-assay-based prediction model could serve as an exploratory indicator of TTR stabilization, we evaluated the relationship between the predicted values and independently collected data on amyloid formation inhibition.

Pearson’s correlation coefficient and Spearman’s rank correlation coefficient were calculated to assess the association between the predicted TTR-binding activity and the amyloid formation inhibition rate. In the pooled dataset comprising all compounds from the four publications, Pearson’s correlation coefficient was r = 0.673, and Spearman’s rank correlation coefficient was ρ = 0.602, indicating a moderate positive correlation between predicted TTR-binding activity and the amyloid formation inhibition rate. However, the correlation coefficients varied across publications (Table 4), indicating that this relationship was not consistently observed in each publication. To further assess whether the association observed in the pooled dataset was explained only by differences among source publications, a source-adjusted regression analysis was performed. The predicted value remained significantly associated with inhibition of amyloid formation after adjustment for source publication, suggesting that the pooled association was not explained only by differences between publications.

The amyloid formation inhibition assays were performed at a compound concentration of 3.6 µM, whereas the Tox24 displacement data used to build the prediction model were collected at 100 µM. Therefore, compounds that showed appreciable TTR-binding at 100 µM may not achieve sufficient target occupancy at the much lower concentration used in the amyloid formation assays. This concentration mismatch may partly explain why some compounds with little or no experimental inhibition yielded relatively high predicted values. Accordingly, directly interpreting the predicted values as amyloid formation inhibitory activity at low concentrations may lead to an overestimation of the inhibitory potential.

In addition, each publication included derivatives based on the same lead compounds; therefore, the present analysis was not based on completely independent compound sets. The results suggest that predicted TTR-binding activity is associated with, but does not fully account for, inhibition of amyloid formation. Therefore, this correlation analysis should be interpreted as providing limited support for the model’s applicability to exploratory screening, rather than as direct biological validation. Screening with this model may serve as a supplementary exploratory approach to prioritize compounds for future amyloid formation inhibition or TTR stabilization assays.

### 3.4. Screening of the PhytoHub Database

The PhytoHub database contains entries with missing SMILES information (383 entries) and missing food-source information (1750 entries) out of a total of 2696 entries (Table S5). The high prevalence of conjugated compounds among entries lacking SMILES information may be due to multiple possible conjugation sites, making it difficult to uniquely define the chemical structure. In addition, compounds described by abstract names cannot be uniquely mapped to specific chemical structures, which may explain the absence of SMILES information.

Furthermore, the high proportion of precursor/metabolite annotations among entries with missing food-source information suggests that these compounds may be organized as metabolic products generated in vivo rather than as compounds directly present in food.

Compounds with Applicability ≥ 0.4 were ranked based on their predicted values for exploratory prioritization. Table 6 lists the 39 compounds with predicted values of 90% or higher. Polyphenolic compounds were particularly abundant among the listed compounds. This trend is consistent with previous studies reporting that polyphenolic compounds can exhibit affinity for the TTR-binding pocket [31,32,33,34,35,36].

The leading candidates identified in this screening, glycitein and daidzein, have been reported to be present in human plasma after soy isoflavone intake [37]. However, daidzein reached only submicromolar levels following a dietary intervention with soy protein. In contrast, glycitein was detected at low concentrations after supplement intake and was not detectable in the soy-protein diet group [37]. These concentrations remain below the reported plasma concentration of TTR. Because the reported plasma concentrations were measured after enzymatic deconjugation and therefore included conjugated forms, the free aglycone concentrations available for direct TTR-binding in vivo may be even lower.

To obtain complementary structural evidence, docking analysis was performed for the selected compounds. Tafamidis was also docked as a positive control (Figures S5 and S6).

The analysis in this study was limited to compounds with clear structural and food-source information. Thus, the resulting dataset may be biased toward parent compounds that are directly present in food, and this should be considered when interpreting the AD and screening results.

In addition, compounds that show TTR-stabilizing activity in vitro may lose activity due to conjugation metabolism. In contrast, some conjugated metabolites, such as the sulfate conjugate of resveratrol, have been reported to retain activity [32]. Thus, future studies should collect structures of conjugates and metabolites, in addition to those of parent compounds, and apply the same prediction model used in the current study to evaluate conditions that more closely reflect in vivo exposure and potential activity.

### 3.5. Study Limitations

#### 3.5.1. Quality of the Training Dataset

One limitation of this study is that the model-predicted values are subject to some uncertainty. The MAE on the test dataset was 15.9, indicating that the model should not be interpreted as providing highly reliable activity values for individual compounds, but rather as a reference index for exploratory candidate prioritization. The model was trained to predict TTR-binding activity values derived from the TTR–ANSA displacement assay. Therefore, the predicted values represent model-based estimates of assay-derived TTR-binding activity values, rather than the displacement rate itself. Thus, values outside the 0–100% range should not be interpreted as actual displacement rates. In addition, the TTR-binding activity values indicate relative ANSA displacement activities calculated by converting the fluorescence values of each compound evaluated at 100 µM using the 0% and 100% activity reference conditions established with T4; they are not direct comparisons with T4 under the same concentration conditions. Furthermore, because only 2D molecular descriptors were used in the present model, stereoisomers were not explicitly differentiated, and the predicted values do not represent activity predictions for specific stereoisomers.

To improve predictive accuracy, it is important to ensure both data quality and appropriate structural coverage. Because specific interactions within the binding pocket may affect TTR-binding, subtle structural changes can produce large differences in activity, i.e., activity cliffs [38]. Capturing broad trends across diverse compounds and detecting local activity changes among structurally related compounds require different types of structural coverage. Therefore, datasets should include not only chemically diverse compounds but also sufficient numbers of structurally related compounds. In the present study, the sample size and structural coverage of the dataset may have been insufficient to fully capture such local structure–activity relationships. This limitation may partly explain why the correlations between predicted TTR-binding activity and inhibition of amyloid formation varied across publications.

In addition, the dataset may contain compounds with low measurement reliability. When analogous compounds differing in terms of only the number of carbon atoms in the main chain were extracted from the training data for comparison with compounds in the test dataset, irregular fluctuations were observed in the measured values, even though a monotonic change with carbon number would be expected (Figure S3). This suggests that the dataset may contain anomalous values originating from measurement failures or related issues. If such inconsistent data are included in the training dataset, the prediction accuracy on the test dataset may be reduced.

Note that the measured values in the present data represent the averages of triplicate measurements, and variability between measurements is expected [17]. Considering this variability, constructing models using data with improved measurement reliability, which can be achieved by increasing the number of replicates to reduce the influence of random errors, could yield improved predictive accuracy.

#### 3.5.2. Regarding Screening

In this study, the screening process was based on predicted TTR-binding activity values and did not directly evaluate actual binding activity. Although predictions within the AD are expected to be more reliable, they do not guarantee quantitative agreement between predicted and measured values. In addition, the present screening workflow has not yet been sufficiently validated using external experimental data for the screened compounds. Therefore, further experimental validation using assays of TTR-binding, tetramer stabilization, and inhibition of amyloid formation will be necessary to confirm the predicted activities and evaluate the utility of this prioritization approach.

Furthermore, this study did not consider pharmacokinetic factors, e.g., compound content in foods, bioavailability, blood kinetics, or competition with albumin. In particular, polyphenols are known to undergo degradation by gut microbiota and conjugation metabolism in vivo, and they are present in the blood mainly as metabolites [39]. Thus, the predicted values for the parent compounds evaluated in this study do not necessarily directly reflect actual TTR-binding capacity or stabilization activity in vivo. Accordingly, the results should be interpreted as an exploratory prioritization of compounds with potential relevance to TTR stabilization, and future studies should include experimental validation of candidate compounds, as well as evaluations of metabolites and pharmacokinetic properties.

## 4. Materials and Methods

The model construction and statistical analyses in this study were performed in a Python environment (version 3.8.0).

### 4.1. Dataset for Model Construction

The train, leaderboard, and test data used for model construction were obtained from OCHEM (https://ochem.eu/static/challenge-data.do, accessed on 29 May 2025) and were originally provided as part of the 2024 Tox24 Challenge.

The Tox24 Challenge is a competition to evaluate advances in computational methods for predicting the in vitro activity of chemical compounds. Each data entry in the complete data package included the compound’s SMILES structure and experimentally measured TTR-binding activity [18]. The assay values in this data package were generated using the TTR–ANSA fluorescence assay, which utilizes the change in fluorescence intensity when a compound displaces the fluorescent probe ANSA bound to TTR. The compound activity was calculated based on a T4 reference scale, with the high-concentration T4 condition (1.8 µM) set to 100% activity and the low-concentration T4 condition (0.0067 µM) set to 0% activity. Each compound was measured at a single concentration of 100 µM, and the TTR-binding activity was calculated from the resulting fluorescence intensity [17]. Therefore, these values should be interpreted as relative ANSA displacement activities defined by the T4 reference conditions, rather than as direct comparisons with T4 at the same concentration.

In this study, the train (N = 1012) and leaderboard (N = 200) datasets were used to train the model. Because the combined dataset contained two entries with the same SMILES, these entries were merged into a single record by taking the median TTR-binding activity, resulting in a final training dataset of 1211 unique compounds. The test (N = 300) dataset was used to evaluate the model’s performance.

### 4.2. Salt/Complex Processing and Molecular Descriptor Calculation

For SMILES notation recorded as salts or complexes, processing was performed to extract only the active moiety. SMILES data were split into fragments using periods as delimiters, and the main component was selected from each set of fragments. In this study, the fragment with the largest number of atoms was extracted as the main component. Then, charge neutralization was applied to the extracted structure, and canonical SMILES notation was regenerated using RDKit (version 2024.03.2). Details about the processing procedure are shown in Figure S4.

Furthermore, molecular descriptors were calculated using Mordred (version 1.2.0). In this study, only 2D descriptors were calculated without generating 3D structures from SMILES (ignore_3D = True). Because this model used only 2D descriptors, stereochemical information, such as chiral centers and E/Z isomerism, was not explicitly considered. Therefore, the model may not distinguish stereoisomers that share the same two-dimensional connectivity, and the predicted values should be interpreted as structure-based prioritization scores rather than stereoisomer-specific activity predictions. Descriptors containing missing values, those with zero variance, and duplicate descriptors were excluded. Three input formats were defined for molecular descriptors, i.e., (a) using only descriptors calculated from structures without salt/complex processing, (b) using only descriptors from structures after salt/complex processing, and (c) integrating descriptors from both preprocessing and postprocessing structures. In format (b), salt/complex processing led to structural duplication among some compounds; therefore, duplicate entries corresponding to the same compound were merged, because retaining such duplicates may promote overfitting and reduce generalization performance. In this case, the activity value was represented by the median. As a result, the number of training compounds decreased from 1211 to 1186. Detailed lists of the descriptors and excluded items for each input format are provided in Tables S8–S10.

### 4.3. Model Construction

The model was constructed using LightGBM (version 3.3.5) in a two-stage process comprising feature selection and hyperparameter optimization. Here, feature selection was performed using fivefold CV, where the data were divided into five subsets: one for validation and four for training in each fold. For each fold, a LightGBM model was trained on the training dataset, and feature importance was calculated based on the number of splits. Hyperparameters were fixed during this stage. The obtained importance scores were averaged across folds, and the features with a mean importance greater than 0 were retained. All subsequent training and evaluation processes used the selected feature set. The feature selection results for each input format are shown in Tables S8–S10.

Hyperparameter optimization was performed in stages. First, Bayesian optimization using Optuna (version 4.0.0) was applied to identify the optimal combination of learning_rate, num_leaves (tree complexity), min_data_in_leaf, min_sum_hessian_in_leaf (leaf constraints), bagging_fraction, feature_fraction (subsampling parameters), lambda_l1, and lambda_l2 (regularization terms). Early stopping was applied during this stage, and training was terminated when the validation metric no longer improved. Next, seven candidate values for the number of iterations (n_estimators) were set by varying the optimal iteration count obtained in the previous stage by several hundred in both directions, and the final value was determined via grid search. Finally, the mean prediction accuracy across fivefold CV was compared for the input formats (a), (b), and (c), and the format that obtained the highest performance was selected. The final model was then constructed using the entire training dataset based on the selected format. The search ranges and determined hyperparameters are shown in Table S11.

### 4.4. Final Model Evaluation

RMSE was used as the evaluation metric, calculated as the square root of the mean squared error between predicted and measured values. Note that the RMSE is scale-dependent on the objective variable; thus, the *R^2^* was also calculated as a supplementary metric. In addition, the MAE was calculated to provide an interpretable estimate of the average prediction error.

(2) $$\mathrm{RMSE}=\sqrt{\frac{1}{N}\sum_{i=1}^{N}{\left(y_{i}-{\hat{y}}_{i}\right)}^{2}}$$

(3) $$\mathrm{MAE}=\frac{1}{N}\sum_{i=1}^{N}\left|y_{i}-{\hat{y}}_{i}\right|$$

To evaluate the reliability of the predictions, an Applicability value was calculated for each compound based on its distance from the training dataset’s chemical space, following the AD approach used in the reference model [40]. Molecular descriptors were normalized based on the training dataset, and the same transformation was applied to the test dataset. For each test sample, the Euclidean distances to the five nearest neighbors in the training dataset were calculated, and an index based on the log-transformed geometric mean of these distances was used to determine the Applicability value. Based on the subsequent evaluation of the relationship between Applicability and prediction error, compounds with an Applicability value of 0 were considered outside the AD in this study.

### 4.5. Model Interpretation by SHAP

The SHAP method quantitatively evaluates the contribution of each feature to model predictions. The explanatory model is expressed as follows:

(4) $$f\left(x\right)=g\left(x^{\prime }\right)=\phi_{0}+\sum_{i=1}^{M}\phi_{i}x_{i}^{\prime }$$

where $\phi_{0}$, $\phi_{i}$, and $x_{i}^{\prime }$ denote the baseline value, the contribution of each feature, and the presence or absence of feature i in the simplified input, respectively. The contribution of each feature is defined as a Shapley value [23].

In this study, the SHAP values were calculated using the training dataset for model interpretation. For missing features, the influence was evaluated by marginalization using the training dataset as the background distribution. Then, feature importance was evaluated using the SHAP values, and the top 10 features with the largest contributions were visualized.

### 4.6. Literature on TTR Amyloid Formation Inhibition Assays

To investigate whether the binding activity predicted by the model developed in this study is associated with experimentally reported inhibition of TTR amyloid formation, compounds and assay data were collected from four publications reporting TTR amyloid formation inhibition assays: Klabunde et al. (2000), Oza et al. (2002), Adamski-Werner et al. (2004), and Palaninathan et al. (2009) [24,25,26,27].

Klabunde et al. (2000) evaluated the amyloid formation rate of wild-type TTR at concentrations of 7.2, 3.6, and 1.8 µM for 12 compounds, primarily nonsteroidal anti-inflammatory drugs [24]. Oza et al. (2002) evaluated 13 diclofenac analogs at 3.6 µM using wild-type and mutant TTR (V30M and L55P) [25]. Adamski-Werner et al. (2004) evaluated 20 compounds, primarily diflunisal analogs, at 7.2 and 3.6 µM for their effects on the amyloid formation rate against wild-type TTR [26]. Palaninathan et al. (2009) evaluated 29 compounds, primarily β-aminoxypropionic acid derivatives, at 3.6 µM for their ability to inhibit amyloid formation in wild-type TTR [27].

Among the assay conditions described in these publications, data from wild-type TTR assays performed at final concentrations of 3.6 µM TTR and 3.6 µM inhibitor were used in this study. Under these conditions, the experimental procedures were generally consistent across the source studies. WT-TTR in phosphate buffer containing KCl and ethylenediaminetetraacetic acid was combined with each compound dissolved in dimethyl sulfoxide and preincubated for 30 min. Then, fibril formation was induced by acidification with acetate buffer, followed by incubation at 37 °C for 72 h without agitation. After incubation, fibril formation was measured by turbidity or optical density and reported relative to the corresponding inhibitor-free WT-TTR control, defined as 100%. Differences in preincubation temperature, acidification pH, and measurement wavelength are summarized in Supplementary Table S12.

In each publication, amyloid formation and inhibition rates were defined relative to amyloid formation in the control without an inhibitor. To unify these metrics for correlation analysis, amyloid formation rates were converted to inhibition rates by subtracting them from 100%, and the dataset was constructed accordingly (Table S4). SMILES were not provided in the publications; therefore, the chemical structures were drawn using ChemDraw (Version 25.0.2.14) to obtain SMILES for each compound. Predictions and AD assessment were then performed on the compiled dataset. Compounds classified as outside the AD were excluded from the subsequent correlation analysis.

Pearson’s correlation coefficient and Spearman’s rank correlation coefficient were calculated to assess the association between predicted values and amyloid formation inhibition rates. Additionally, source-adjusted regression analysis was performed using the source publication as a categorical covariate.

### 4.7. Correlation Analysis

To evaluate the association between the predicted TTR-binding activity and amyloid formation inhibition rate, both Pearson’s correlation coefficient and Spearman’s rank correlation coefficient were calculated. Pearson’s correlation coefficient was calculated to assess the linear association between the predicted values and inhibition rates. In contrast, Spearman’s rank correlation coefficient was calculated to assess the rank-based monotonic association.

These correlation coefficients were calculated for the integrated dataset, including all compounds from the four publications, and separately for each publication to assess publication-specific variability in the association. To examine the influence of Applicability on this association, the same correlation analyses were performed after applying different Applicability thresholds.

### 4.8. PhytoHub Database

PhytoHub version 1.4 was used to screen food-derived compounds [28]. The “Chemical Name,” “Phytochemical family,” “Smiles,” “Food sources,” and “Metabolites/Precursor” columns were extracted for analysis. Because SMILES and food-source information were not available for all entries, the annotation status of the “Metabolites/Precursor” column was examined, and entries lacking either “Smiles” or “Food sources” information were excluded to construct the analytical dataset. The compounds in the analytical dataset were classified based on the “Phytochemical family” column. Entries classified as “Miscellaneous phytochemicals” and “Not Available” were combined as “Others” because their classification could not be uniquely determined.

Predictions and Applicability assessment were then performed, and compounds with Applicability values of 0.4 or higher were ranked according to their predicted values.

Molecular docking was performed using GNINA version 1.3.1, with the TTR structure from PDB ID 3TCT used as the receptor [41]. The binding region was defined according to the tafamidis-binding site, and the top-ranked pose was retained for each compound. Tafamidis was included as a positive control.

## 5. Conclusions

ATTRwt is a progressive disease characterized by amyloid deposition in the heart, which is frequently observed in elderly individuals [15]. However, non-pharmacological strategies for ATTRwt have not been sufficiently established. In this study, we developed a TTR-binding activity prediction model and applied it to the exploratory prioritization of food-derived compounds registered in PhytoHub, a phytochemical database.

The model was developed to predict TTR-binding activity using TTR–ANSA displacement assay data. Applying the model to the PhytoHub dataset prioritized compounds with high predicted TTR-binding activity, many of which were classified as polyphenols.

The model demonstrated predictive performance on the independent test dataset corresponding to an estimated seventh-place ranking among the 78 teams participating in the Tox24 Challenge, supporting its utility for the exploratory prioritization of compounds within its Applicability Domain. Predicted TTR-binding activity was also positively correlated with experimentally reported inhibition of amyloid formation; however, the strength of this relationship varied across the literature datasets, indicating that predicted binding activity should not be interpreted as a direct measure of TTR stabilization or amyloid inhibition.

The significance of this study lies in providing an in silico method for prioritizing food-derived compounds for future experimental evaluation. However, the model developed in this study does not directly predict TTR stabilization activity; rather, it assesses TTR–ANSA displacement-based TTR-binding activity. Therefore, further validation using direct TTR-binding, stabilization, and inhibition of amyloid formation assays is required to determine whether the prioritized compounds can bind to or stabilize TTR.

Overall, the results of this study should be interpreted as hypothesis-generating findings for future experimental research rather than as evidence for ATTRwt prevention or dietary intervention. Multifaceted investigations, including experimental validation and pharmacokinetic evaluation, are required in future studies.

## Figures and Tables

**Table 1:** Progression of descriptor counts through preprocessing stages, including missing value removal and zero-variance filtering, followed by feature selection based on feature importance.

Input Format	Number of Descriptors					
	Total	Preprocessing			Feature Selection	
		Missing Values	Zero Variance	After Filtering	Importance = 0	Final
**(a)**	**1613**	**884**	**119**	**610**	**167**	**443**
**(b)**	**1613**	**729**	**123**	**761**	**239**	**522**
**(c)**	**3226**	**1613**	**242**	**1371**	**551**	**820**

**Table 2:** Comparison of predictive accuracy (RMSE) across input formats in fivefold CV.

Input Format	RMSE						
	Fold_1	Fold_2	Fold_3	Fold_4	Fold_5	Mean	Std
**(a)**	**22.53**	**23.17**	**24.79**	**23.82**	**22.81**	**23.42**	**0.90**
**(b)**	**22.37**	**24.00**	**23.04**	**23.59**	**23.23**	**23.25**	**0.61**
**(c)**	**22.17**	**22.39**	**24.34**	**22.89**	**22.33**	**22.82**	**0.89**

**Table 3:** Descriptions of the top 10 Mordred molecular descriptors identified as most important by SHAP analysis.

Descriptor	Mordred Official Description
**SLogP**	**Wildman–Crippen LogP**
**SlogP_VSA11**	**MOE logP VSA Descriptor 11 (0.50 ≤ x < 0.60)**
**AETA_beta_ns_d**	**averaged nonsigma contribution to valence electron mobile count**
**ETA_dEpsilon_D**	**ETA delta epsilon (type: D)**
**ATSC1i**	**centered Moreau–Broto autocorrelation of lag 1 weighted by ionization potential**
**ETA_dEpsilon_B**	**ETA delta epsilon (type: B)**
**BertzCT**	**Bertz CT**
**SMR_VSA3**	**MOE MR VSA Descriptor 3 (1.82 ≤ x < 2.24)**
**NaasC**	**number of aasC**
**piPC2**	**2-ordered pi-path count (log scale)**

**Table 4:** Pearson’s correlation coefficients and Spearman’s rank correlation coefficients for each publication and all compounds combined.

Source	N_compounds	Pearson’s r	Spearman’s ρ
**Klabunde et al., *Nat. Struct. Biol.* (2000)** [24]	**10**	**0.114**	**−0.030**
**Oza et al., *J. Med. Chem.* (2002)** [25]	**13**	**0.801**	**0.682**
**Adamski-Werner et al., *J. Med. Chem.* (2004)** [26]	**26**	**−0.119**	**−0.073**
**Palaninathan et al., *PLoS One* (2009)** [27]	**29**	**0.525**	**0.411**
**Pooled**	**78**	**0.673**	**0.602**

**Table 5:** Source-adjusted regression analysis of predicted TTR–ANSA displacement activity and observed amyloid inhibition.

Model	N	Explanatory Variable	β	95% CI	*p*-Value	*R^2^*
**Simple regression**	**78**	**Predicted value**	**0.780**	**0.583–0.976**	**1.55 × 10^−11^**	**0.452**
**Source-adjusted regression**	**78**	**Predicted value**	**0.553**	**0.269–0.837**	**2.25 × 10^−4^**	**0.550**

**Table 6:** Exploratory candidate compounds with predicted values exceeding 90%.

Chemical Name	Phytochemical Family	Predicted Value	Applicability
**Glycitein**	**Polyphenols**	**104.67**	**0.62**
**Daidzein**	**Polyphenols**	**104.02**	**0.65**
**2′-Hydroxydaidzein**	**Polyphenols**	**102.79**	**0.64**
**Cyanidin**	**Polyphenols**	**101.71**	**0.48**
**Genistein**	**Polyphenols**	**101.69**	**0.67**
**Hispidulin**	**Polyphenols**	**101.55**	**0.59**
**Apigenin**	**Polyphenols**	**101.42**	**1.00**
**Calycosin**	**Polyphenols**	**101.32**	**0.60**
**Biochanin A**	**Polyphenols**	**100.11**	**1.00**
**Herbacetin**	**Polyphenols**	**99.89**	**0.43**
**Pratensein**	**Polyphenols**	**99.68**	**0.60**
**Quercetin**	**Polyphenols**	**99.31**	**0.46**
**Resveratrol (trans-)**	**Polyphenols**	**98.69**	**0.62**
**Resveratrol (cis-)**	**Polyphenols**	**98.69**	**0.62**
**Luteolin**	**Polyphenols**	**98.05**	**0.61**
**Betuletol**	**Polyphenols**	**97.79**	**0.43**
**Isorhamnetin**	**Polyphenols**	**97.77**	**0.48**
**Rhapontigenin**	**Polyphenols**	**97.42**	**0.55**
**Pinosylvin**	**Polyphenols**	**97.40**	**0.67**
**Prunetin**	**Polyphenols**	**97.40**	**0.65**
**Isorhapontigenin**	**Polyphenols**	**97.30**	**0.56**
**Petunidin**	**Polyphenols**	**97.26**	**0.42**
**Kaempferol**	**Polyphenols**	**96.88**	**0.58**
**Pelargonidin**	**Polyphenols**	**96.76**	**0.58**
**1,3-trans-Tetrahydroxyphenylindan**	**Others**	**96.59**	**0.50**
**1,3-cis-Tetrahydroxyphenylindan**	**Others**	**96.59**	**0.50**
**Piceatannol (cis-)**	**Polyphenols**	**95.17**	**0.54**
**Piceatannol**	**Polyphenols**	**95.17**	**0.54**
**desmethylxanthohumol**	**Others**	**95.02**	**0.42**
**Emodin**	**Others**	**94.92**	**0.46**
**Oxyresveratrol (cis-)**	**Polyphenols**	**93.61**	**0.54**
**Oxyresveratrol**	**Polyphenols**	**93.56**	**0.53**
**Dihydro-resveratrol**	**Polyphenols**	**93.14**	**0.65**
**Formononetin**	**Polyphenols**	**92.92**	**0.64**
**Liquiritigenin**	**Polyphenols**	**92.18**	**0.63**
**Dihydro-piceatannol**	**Polyphenols**	**91.59**	**0.55**
**Chrysophanol**	**Others**	**91.06**	**0.50**
**Naringenin**	**Polyphenols**	**90.90**	**0.64**
**Hesperetin**	**Polyphenols**	**90.25**	**0.53**

## Data Availability

Data are contained within the article and Supplementary Materials. The source code used for data preprocessing, model development, evaluation, and compound screening is available in the GitHub repository at https://github.com/git-uma/TTR-binding-activity.git (accessed on 17 July 2026).

## Supplementary Materials

The following supporting information can be downloaded at https://www.mdpi.com/article/10.3390/ph19081295/s1: Figure S1: Relationship between predicted and experimental inhibition values across different Applicability thresholds. Points represent compounds from different data sources, and crosses represent compounds excluded at each threshold. Pearson’s r, Spearman’s ρ, and the number of remaining compounds are shown in each panel. Figure S2: Changes in Pearson’s correlation coefficient, Spearman’s rank correlation coefficient, and the number of remaining compounds across Applicability thresholds. Pearson’s r and Spearman’s ρ are shown in blue and orange, respectively, while the number of compounds retained at each threshold is shown in green. Figure S3: Example of a compound with suspected measurement failure. For nonanal compounds included in the test dataset, analogous compounds differing only in the number of main-chain carbon atoms were extracted from the training dataset and compared. The measured value of octanal did not fall within the range bounded by the measured values of heptanal and nonanal. Figure S4: Pseudocode for salt/complex processing. Figure S5: Predicted binding poses of selected food-derived compounds and tafamidis in the TTR thyroxine-binding channel. For each compound, the chemical name, predicted TTR-binding activity (%), and two GNINA outputs—the Vina affinity score (kcal/mol) and CNN-predicted affinity—are presented. Ligands are displayed with the surrounding binding-pocket residues and molecular surface. Figure S6: Superposition of the crystallographic tafamidis pose (yellow) and the docked pose (gray) in the TTR thyroxine-binding channel. Because the crystal structure contained two symmetry-related tafamidis poses, the docked pose was compared with both reference poses, and the minimum symmetry-corrected heavy-atom root mean square deviation (RMSD) was reported. The figure shows the reference pose that gave the lower RMSD. The minimum RMSD was 0.867 Å, indicating that the docking protocol successfully reproduced the crystallographic binding mode and supporting the reliability of the predicted ligand poses. Table S1: Model construction dataset containing SMILES, TTR-binding activity (%), measurement units, and dataset assignment (train/leaderboard datasets). Table S2: Test dataset with predicted TTR-binding activity values, $s_{i}$ values calculated as the log-transformed geometric mean of distances to the five nearest neighbors, Applicability values, and AD classification results. Table S3: Experimental conditions for the TTR amyloid aggregation inhibition assays used for dataset construction. Table S4: Amyloid formation inhibition assay dataset compiled from four literature sources, including SMILES, % inhibition, predicted TTR-binding activity values, $s_{i}$ values, Applicability values, and AD classification results. Table S5: Complete PhytoHub dataset extracted for analysis, including “Chemical Name,” “Phytochemical family,” “Smiles,” “Food sources,” and “Metabolites/Precursor.” Table S6: PhytoHub analytical dataset after exclusion of entries with missing “Smiles” or “Food sources” information. Table S7: PhytoHub analytical dataset with predicted TTR-binding activity values, $s_{i}$ values, Applicability values, and AD classification results. Table S8: Detailed descriptor list and preprocessing/feature selection results for input format (a) using descriptors calculated from structures without salt/complex processing. Table S9: Detailed descriptor list and preprocessing/feature selection results for input format (b) using descriptors calculated from structures after salt/complex processing. Table S10: Detailed descriptor list and preprocessing/feature selection results for input format (c), integrating descriptors from both preprocessing and postprocessing structures. Table S11: Search ranges and selected hyperparameters for the LightGBM models constructed based on each input format. Table S12: Assay conditions reported in the four source publications.

## Abbreviations

The following abbreviations are used in this manuscript:

AD	Applicability Domain
ATTR	Transthyretin amyloidosis
CI	Confidence interval
CV	Cross-validation
MAE	Mean absolute error
RMSE	Root mean square error
SHAP	SHapley Additive exPlanations
SD	Standard deviation
SMILES	Simplified Molecular Input Line Entry System
TTR	Transthyretin

## References

[1] Ando Y., Adams D., Benson M.D., Berk J.L., Planté-Bordeneuve V., Coelho T., Conceição I., Ericzon B.-G., Obici L., Rapezzi C. (2022). Guidelines and New Directions in the Therapy and Monitoring of ATTRv Amyloidosis. Amyloid.

[2] Saraiva M.J.M., Costa P.P., Goodman D.S. (1985). Biochemical Marker in Familial Amyloidotic Polyneuropathy, Portuguese Type. Family Studies on the Transthyretin (Prealbumin)-Methionine-30 Variant. J. Clin. Investig..

[3] Jacobson D.R., Pastore R.D., Yaghoubian R., Kane I., Gallo G., Buck F.S., Buxbaum J.N. (1997). Variant-Sequence Transthyretin (Isoleucine 122) in Late-Onset Cardiac Amyloidosis in Black Americans. N. Engl. J. Med..

[4] Westermark P., Sletten K., Johansson B., Cornwell G.G. (1990). Fibril in Senile Systemic Amyloidosis Is Derived from Normal Transthyretin. Proc. Natl. Acad. Sci. USA.

[5] Magalhães J., Eira J., Liz M.A. (2021). The Role of Transthyretin in Cell Biology: Impact on Human Pathophysiology. Cell. Mol. Life Sci..

[6] Hammarström P., Jiang X., Hurshman A.R., Powers E.T., Kelly J.W. (2002). Sequence-Dependent Denaturation Energetics: A Major Determinant in Amyloid Disease Diversity. Proc. Natl. Acad. Sci. USA.

[7] Lai Z., Colón W., Kelly J.W. (1996). The Acid-Mediated Denaturation Pathway of Transthyretin Yields a Conformational Intermediate That Can Self-Assemble into Amyloid. Biochemistry.

[8] Colon W., Kelly J.W. (1992). Partial Denaturation of Transthyretin Is Sufficient for Amyloid Fibril Formation in Vitro. Biochemistry.

[9] Anan I. (2025). Advances in the Treatment of Transthyretin Amyloidosis. eGastroenterology.

[10] Kazi D.S., Bellows B.K., Baron S.J., Shen C., Cohen D.J., Spertus J.A., Yeh R.W., Arnold S.V., Sperry B.W., Maurer M.S. (2020). Cost-Effectiveness of Tafamidis Therapy for Transthyretin Amyloid Cardiomyopathy. Circulation.

[11] Fukunari A., Matsushita H., Furukawa T., Matsuzaki H., Tanaka H., Ogawa Y., Sugimura Y., Inoue F., Ueda M., Ando Y. (2024). Arginine: A Potential Prophylactic Supplement for Transthyretin Amyloidosis. Biochem. Biophys. Res. Commun..

[12] Miyata M., Sato T., Kugimiya M., Sho M., Nakamura T., Ikemizu S., Chirifu M., Mizuguchi M., Nabeshima Y., Suwa Y. (2010). The Crystal Structure of the Green Tea Polyphenol (−)-Epigallocatechin Gallate−Transthyretin Complex Reveals a Novel Binding Site Distinct from the Thyroxine Binding Site. Biochemistry.

[13] Kristen A.V., Lehrke S., Buss S., Mereles D., Steen H., Ehlermann P., Hardt S., Giannitsis E., Schreiner R., Haberkorn U. (2012). Green Tea Halts Progression of Cardiac Transthyretin Amyloidosis: An Observational Report. Clin. Res. Cardiol..

[14] Ciccone L., Tonali N., Nencetti S., Orlandini E. (2020). Natural Compounds as Inhibitors of Transthyretin Amyloidosis and Neuroprotective Agents: Analysis of Structural Data for Future Drug Design. J. Enzym. Inhib. Med. Chem..

[15] Ueda M., Horibata Y., Shono M., Misumi Y., Oshima T., Su Y., Tasaki M., Shinriki S., Kawahara S., Jono H. (2011). Clinicopathological Features of Senile Systemic Amyloidosis: An Ante- and Post-Mortem Study. Mod. Pathol..

[16] Powers E.T., Amass L., Baylor L., Fernández-Arias I., Riley S., Kelly J.W. (2025). Transthyretin Kinetic Stabilizers for ATTR Amyloidosis: A Narrative Review of Mechanisms and Therapeutic Benefits. Cardiol. Ther..

[17] Eytcheson S.A., Zosel A.D., Olker J.H., Hornung M.W., Degitz S.J. (2024). Screening the ToxCast Chemical Libraries for Binding to Transthyretin. Chem. Res. Toxicol..

[18] Tetko I.V. (2024). Tox24 Challenge. Chem. Res. Toxicol..

[19] Moriwaki H., Tian Y.-S., Kawashita N., Takagi T. (2018). Mordred: A Molecular Descriptor Calculator. J. Cheminform..

[20] Ke G., Meng Q., Finley T., Wang T., Chen W., Ma W., Ye Q., Liu T.-Y. (2017). LightGBM: A Highly Efficient Gradient Boosting Decision Tree. Proceedings of the 31st International Conference on Neural Information Processing Systems.

[21] Eytcheson S.A., Tetko I.V. (2025). Which Modern AI Methods Provide Accurate Predictions of Toxicological End Points? Analysis of Tox24 Challenge Results. Chem. Res. Toxicol..

[22] OECD (2014). Guidance Document on the Validation of (Quantitative) Structure-Activity Relationship [(Q)SAR] Models.

[23] Lundberg S.M., Lee S.-I. (2017). A Unified Approach to Interpreting Model Predictions. arXiv.

[24] Klabunde T., Petrassi H.M., Oza V.B., Raman P., Kelly J.W., Sacchettini J.C. (2000). Rational Design of Potent Human Transthyretin Amyloid Disease Inhibitors. Nat. Struct. Biol..

[25] Oza V.B., Smith C., Raman P., Koepf E.K., Lashuel H.A., Petrassi H.M., Chiang K.P., Powers E.T., Sachettinni J., Kelly J.W. (2002). Synthesis, Structure, and Activity of Diclofenac Analogues as Transthyretin Amyloid Fibril Formation Inhibitors. J. Med. Chem..

[26] Adamski-Werner S.L., Palaninathan S.K., Sacchettini J.C., Kelly J.W. (2004). Diflunisal Analogues Stabilize the Native State of Transthyretin. Potent Inhibition of Amyloidogenesis. J. Med. Chem..

[27] Palaninathan S.K., Mohamedmohaideen N.N., Orlandini E., Ortore G., Nencetti S., Lapucci A., Rossello A., Freundlich J.S., Sacchettini J.C. (2009). Novel Transthyretin Amyloid Fibril Formation Inhibitors: Synthesis, Biological Evaluation, and X-Ray Structural Analysis. PLoS ONE.

[28] Institut National de la Recherche Agronomique PhytoHub. https://phytohub.eu/.

[29] Online Chemical Modeling Environment. https://ochem.eu/static/challenge.do.

[30] Blake C.C.F., Geisow M.J., Oatley S.J., Rérat B., Rérat C. (1978). Structure of Prealbumin: Secondary, Tertiary and Quaternary Interactions Determined by Fourier Refinement at 1.8 Å. J. Mol. Biol..

[31] Trivella D.B.B., dos Reis C.V., Lima L.M.T.R., Foguel D., Polikarpov I. (2012). Flavonoid Interactions with Human Transthyretin: Combined Structural and Thermodynamic Analysis. J. Struct. Biol..

[32] Florio P., Folli C., Cianci M., Rio D.D., Zanotti G., Berni R. (2015). Transthyretin Binding Heterogeneity and Anti-Amyloidogenic Activity of Natural Polyphenols and Their Metabolites. J. Biol. Chem..

[33] Yokoyama T., Ueda M., Ando Y., Mizuguchi M. (2015). Discovery of γ-Mangostin as an Amyloidogenesis Inhibitor. Sci. Rep..

[34] Iakovleva I., Begum A., Pokrzywa M., Walfridsson M., Sauer-Eriksson A.E., Olofsson A. (2015). The Flavonoid Luteolin, but Not Luteolin-7-O-Glucoside, Prevents a Transthyretin Mediated Toxic Response. PLoS ONE.

[35] Ferreira N., Saraiva M.J., Almeida M.R. (2011). Natural Polyphenols Inhibit Different Steps of the Process of Transthyretin (TTR) Amyloid Fibril Formation. FEBS Lett..

[36] Pullakhandam R., Srinivas P.N.B.S., Nair M.K., Reddy G.B. (2009). Binding and Stabilization of Transthyretin by Curcumin. Arch. Biochem. Biophys..

[37] Van Der Velpen V., Hollman P.C., Van Nielen M., Schouten E.G., Mensink M., Van’T Veer P., Geelen A. (2014). Large Inter-Individual Variation in Isoflavone Plasma Concentration Limits Use of Isoflavone Intake Data for Risk Assessment. Eur. J. Clin. Nutr..

[38] Maggiora G.M. (2006). On Outliers and Activity Cliffs—Why QSAR Often Disappoints. J. Chem. Inf. Model..

[39] Gade A., Kumar M.S. (2023). Gut Microbial Metabolites of Dietary Polyphenols and Their Potential Role in Human Health and Diseases. J. Physiol. Biochem..

[40] Kurosaki K., Wu R., Uesawa Y. (2020). A Toxicity Prediction Tool for Potential Agonist/Antagonist Activities in Molecular Initiating Events Based on Chemical Structures. Int. J. Mol. Sci..

[41] McNutt A.T., Francoeur P., Aggarwal R., Masuda T., Meli R., Ragoza M., Sunseri J., Koes D.R. (2021). GNINA 1.0: Molecular Docking with Deep Learning. J. Cheminform..

