# Supplementary material for: Machine Learning-Based Prediction of Transthyretin-Binding Activity and Its Application to Screening Food-Derived Compounds for ATTR Amyloidosis
Source: Pharmaceuticals (Basel). 2026 Aug 16;19(8):1295. doi: 10.3390/ph19081295 (PMC13516772; doi:10.3390/ph19081295)
Supplement: Supplementary file 1 [file pharmaceuticals-19-01295-s001.zip › Supplementary_Figures.pdf]

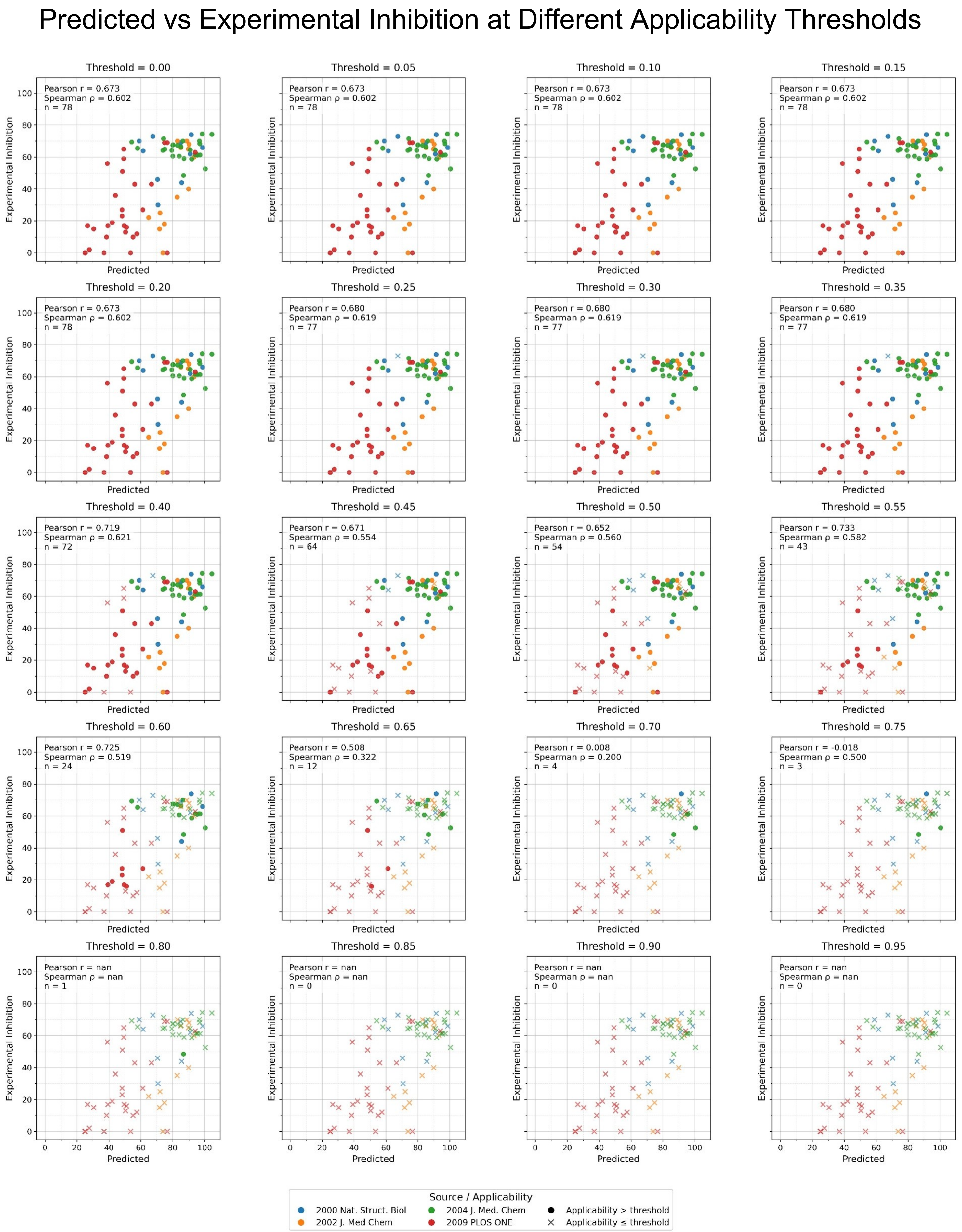

Figure S1. Relationship between predicted and experimental inhibition values across different Applicability thresholds. Points represent compounds from different data sources, and crosses represent compounds excluded at each threshold. Pearson's *r*, Spearman's *ρ*, and the number of remaining compounds are shown in each panel.

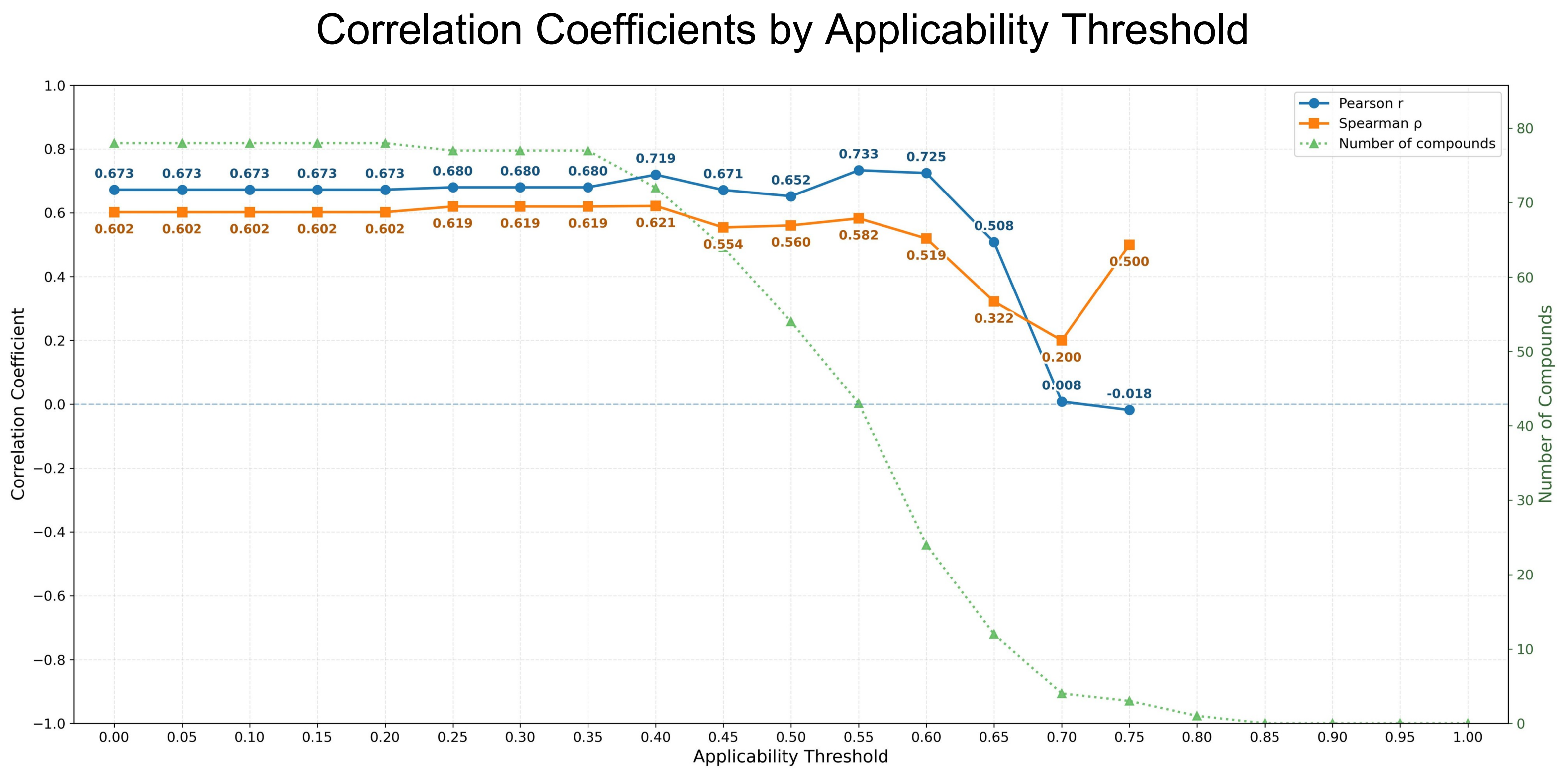

Figure S2. Changes in Pearson's *r*, Spearman's *ρ*, and the number of remaining compounds across Applicability thresholds. Pearson's *r* and Spearman's *ρ* are shown in blue and orange, respectively, while the number of compounds retained at each threshold is shown in green.

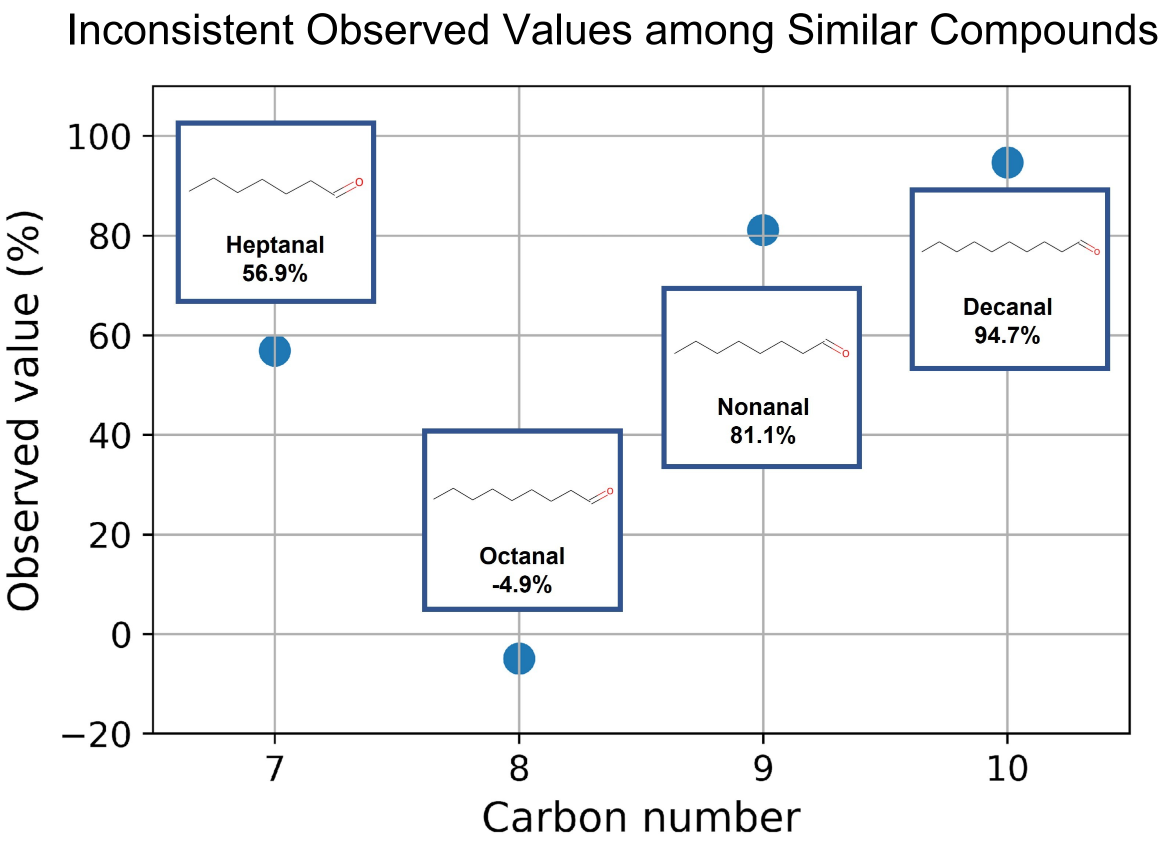

Figure S3. Example of a compound with suspected measurement failure. For nonanal compounds included in the test dataset, analogous compounds differing only in the number of main-chain carbon atoms were extracted from the training dataset and compared. The measured value of octanal did not fall within the range bounded by the measured values of heptanal and nonanal.

Algorithm 1 Desalting and normalization of SMILES strings

Input: A SMILES string s

Output: A normalized SMILES string s'

```
Normalize charge notation in s
    (e.g., convert "++" → "+2")
Ensure that charge symbols are placed at the end of bracket expressions
    (e.g., [O-H] → [OH-])
if s contains multiple fragments separated by "." then
    Split s into fragments {f1, f2, . . . , fn}
    Convert each fragment into a molecular representation
    Select fragment(s) with the largest number of atoms
    if cation-containing fragments exist then
        Retain only those fragments
    end if
    if multiple candidates remain then
        Select the fragment with the highest total mass of cationic elements
    end if
    Replace s with the selected fragment
end if
Apply predefined charge-neutralization rules
    (e.g., for protonated amines, carboxylates, thiolates, etc.)
Convert the processed structure into canonical SMILES
return s′
```

Figure S4. Pseudocode for salt/complex processing.

Predicted Binding Poses in the TTR Thyroxine-Binding Channel

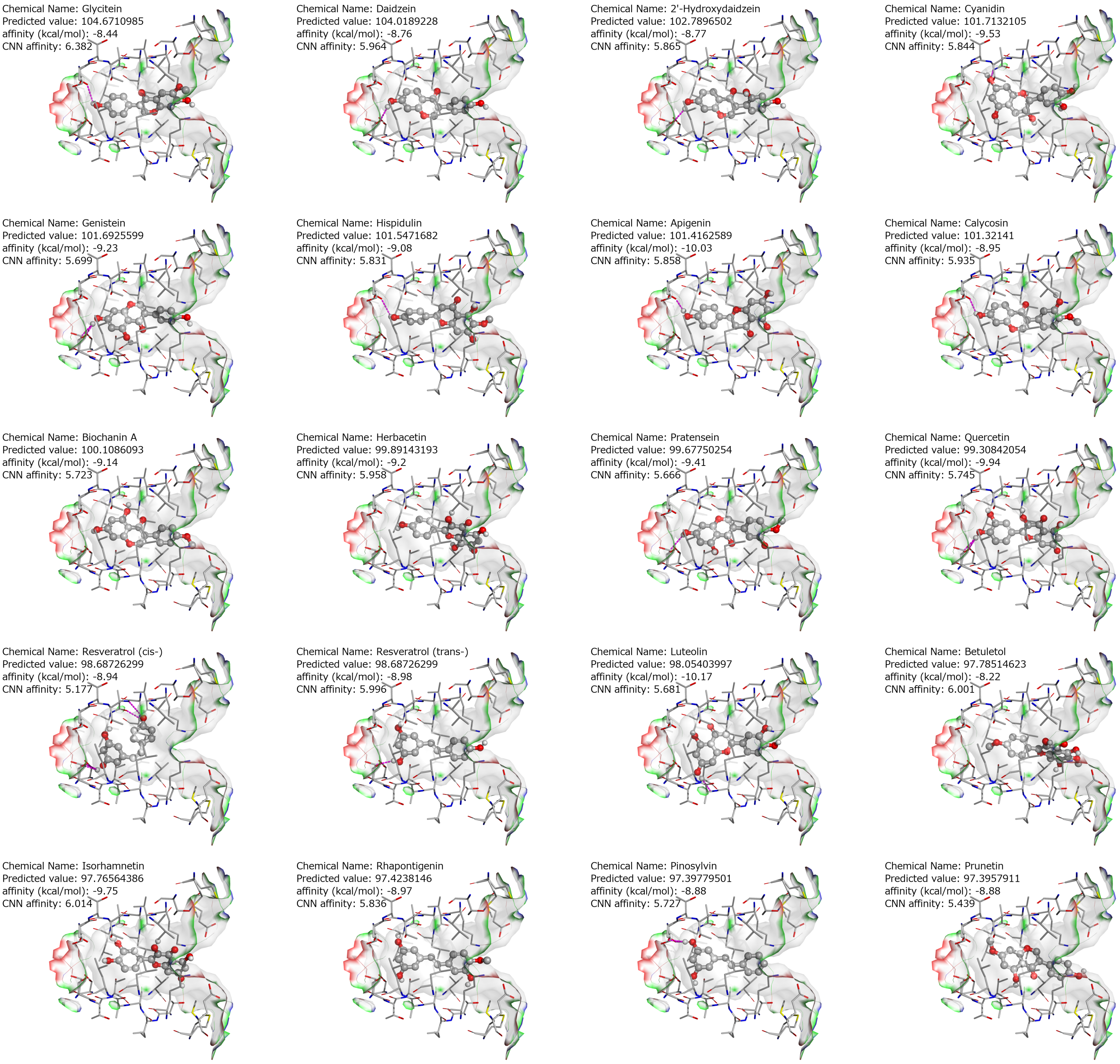

Figure S5. Predicted binding poses of selected food-derived compounds and tafamidis in the TTR thyroxine-binding channel. For each compound, the chemical name, predicted TTR-binding activity (%), and two GNINA outputs (the Vina affinity score [kcal/mol] and CNN-predicted affinity) are presented. Ligands are displayed with the surrounding binding-pocket residues and molecular surface.

Predicted Binding Poses in the TTR Thyroxine-Binding Channel (continued)

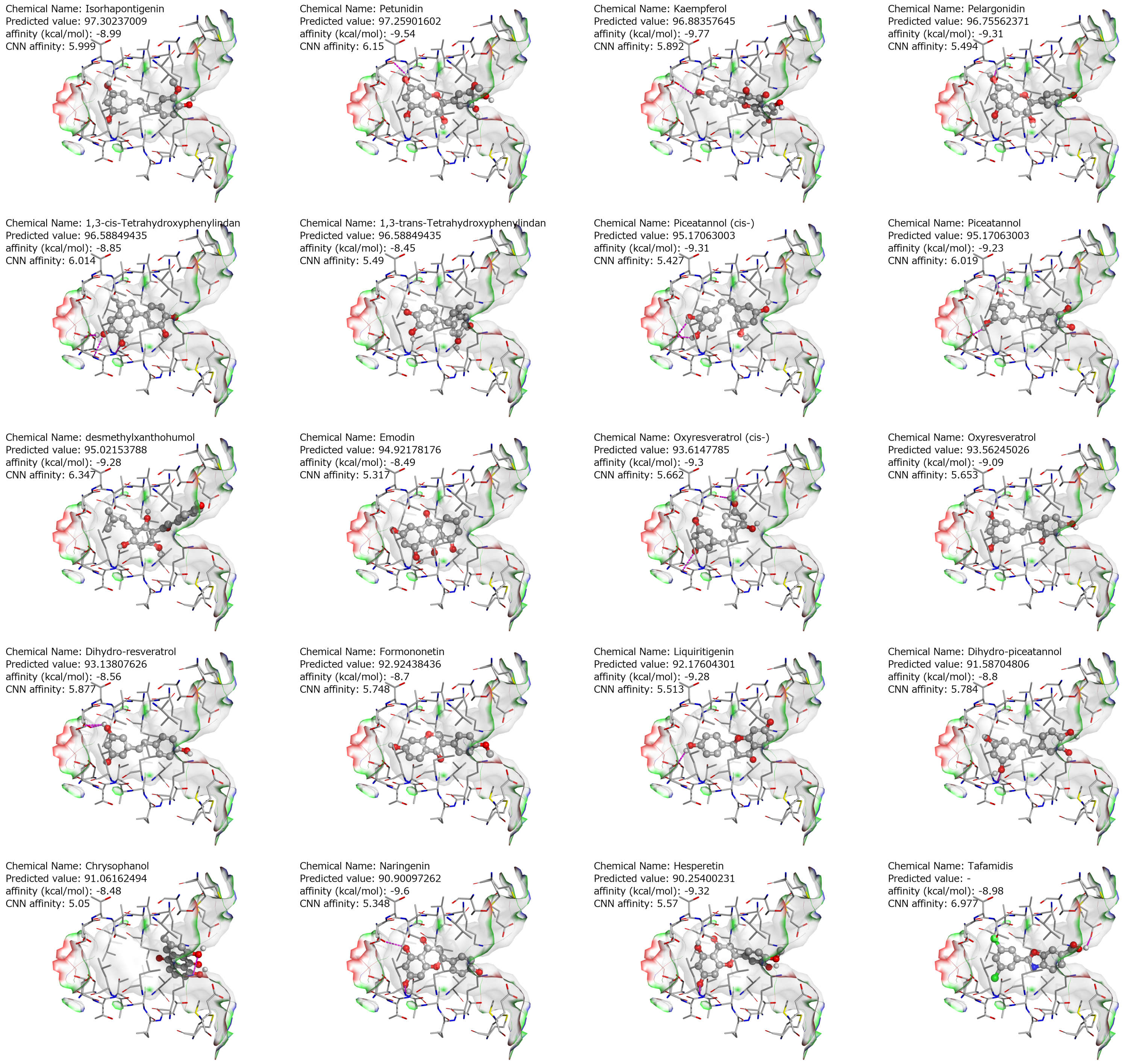

Figure S5 (continued). Predicted binding poses of selected food-derived compounds and tafamidis in the TTR thyroxine-binding channel.

Superposition of Crystallographic and Docked Tafamidis Poses

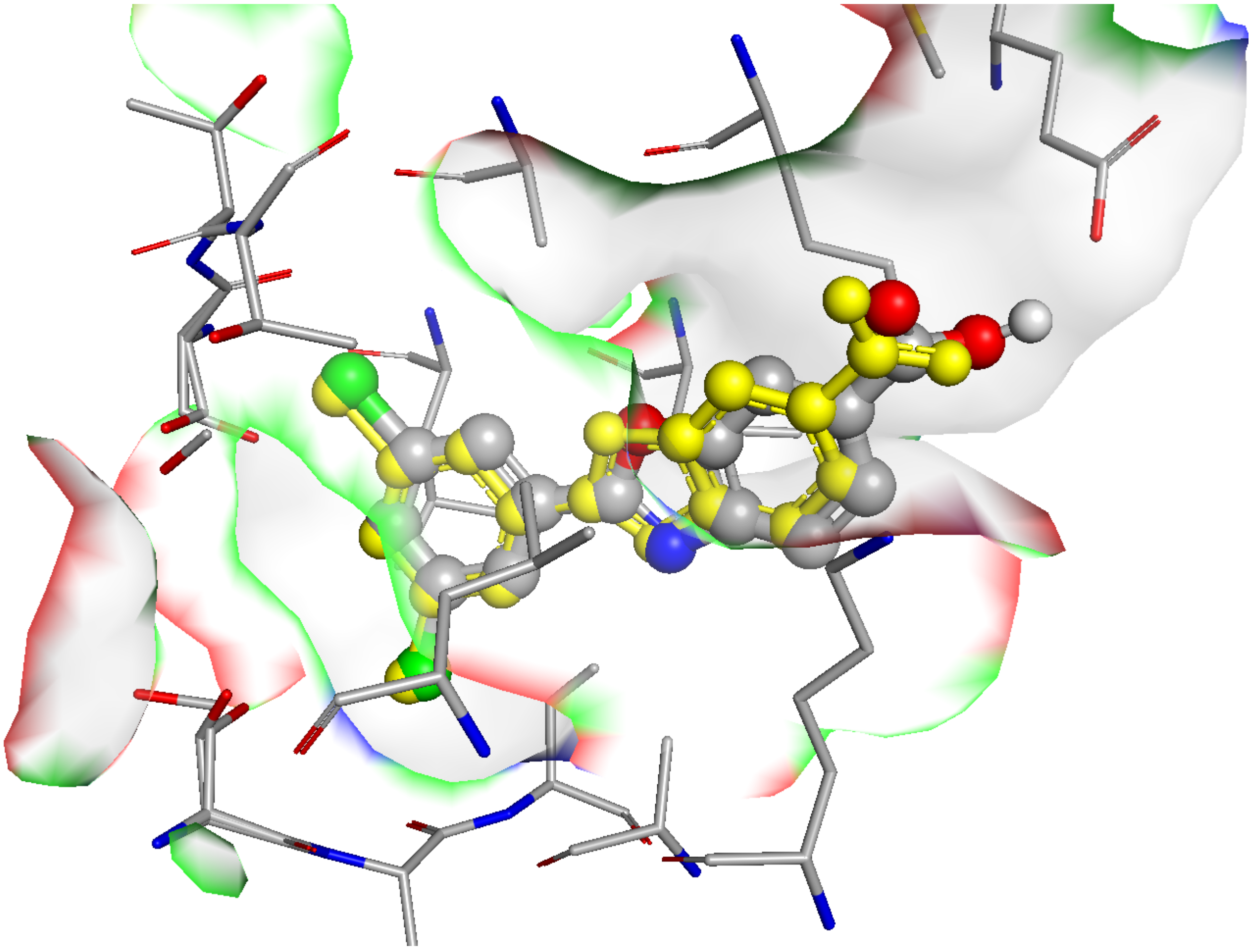

Figure S6. Superposition of the crystallographic tafamidis pose (yellow) and the docked pose (gray) in the TTR thyroxine-binding channel. Because the crystal structure contained two symmetry-related tafamidis poses, the docked pose was compared with both reference poses, and the minimum symmetry-corrected heavy-atom root mean square deviation (RMSD) was reported. The figure shows the reference pose that gave the lower RMSD. The minimum RMSD was 0.867 Å, indicating that the docking protocol successfully reproduced the crystallographic binding mode and supporting the reliability of the predicted ligand poses.
